# Adaptive Resistance in Bacteria Requires Epigenetic Inheritance, Genetic Noise, and Cost of Efflux Pumps

**DOI:** 10.1371/journal.pone.0118464

**Published:** 2015-03-17

**Authors:** Santiago Sandoval Motta, Philippe Cluzel, Maximino Aldana

**Affiliations:** 1 Instituto de Ciencias Físicas, UNAM, Cuernavaca, Morelos, Mexico; 2 FAS Center for Systems Biology, Harvard University, Cambridge, Massachusetts, United States of America; KRIBB, KOREA, REPUBLIC OF

## Abstract

Adaptive resistance emerges when populations of bacteria are subjected to gradual increases of antibiotics. It is characterized by a rapid emergence of resistance and fast reversibility to the non-resistant phenotype when the antibiotic is removed from the medium. Recent work shows that adaptive resistance requires epigenetic inheritance and heterogeneity of gene expression patterns that are, in particular, associated with the production of porins and efflux pumps. However, the precise mechanisms by which inheritance and variability govern adaptive resistance, and what processes cause its reversibility remain unclear. Here, using an efflux pump regulatory network (EPRN) model, we show that the following three mechanisms are essential to obtain adaptive resistance in a bacterial population: 1) intrinsic variability in the expression of the EPRN transcription factors; 2) epigenetic inheritance of the transcription rate of EPRN associated genes; and 3) energetic cost of the efflux pumps activity that slows down cell growth. While the first two mechanisms acting together are responsible for the emergence and gradual increase of the resistance, the third one accounts for its reversibility. In contrast with the standard assumption, our model predicts that adaptive resistance cannot be explained by increased mutation rates. Our results identify the molecular mechanism of epigenetic inheritance as the main target for therapeutic treatments against the emergence of adaptive resistance. Finally, our theoretical framework unifies known and newly identified determinants such as the burden of efflux pumps that underlie bacterial adaptive resistance to antibiotics.

## Introduction

It has been well-established that various species of bacteria, including *E*. *coli*, *S*. *enterica* and *P*. *aeruginosa*, exhibit resistance when they are exposed to successive steps of increasing concentration of antibiotics [1–9]. This procedure, repeated several times, very quickly yields populations with high levels of resistance [1, 3–8, 9]. Another important observation is that this resistance is highly reversible. When the antibiotic is removed from the environment, the population becomes sensitive again after a few generations [1, 3–8, 9]. Although the field remains significantly unexplored, this temporal ability to cope with antibiotics is often referred to as adaptive resistance [10]. As there is no universally accepted definition of adaptive resistance, we will use the one given by Fernandez & Hancock: “a temporary increase in the ability of a bacterium to survive an antibiotic insult due to alterations in gene and/or protein expression as a result of an exposure to an environmental trigger […] and usually reverts upon the removal of the inducing condition.” [10].

Experimental observations indicate that high phenotypic variability, in an isogenic population, can often be used as a bet-hedging strategy that permits the selection of resistant cells even at low antibiotic concentrations [1, 3, 11]. This observation was recently corroborated by experiments showing that variability in expression of the Mar/AcrAB efflux pump system in *E*. *coli* is correlated with the appearance of adaptive resistance to some antibiotics such as nalidixic acid [1, 3, 5, 9, 10, 11].

This variability cannot be governed by mutations alone, as the survival rates observed in these experiments are too high compared to what would be expected by mutations. For instance, it is estimated that the probability to find a *P*. *aeruginosa* mutant with stable resistance to low-level Quinolone is about 1.2×10^−6^ to 4×10^−10^, whereas Adam et al. found survival rates as large as 20% of the population after the first antibiotic exposure [1].

Moreover, the reversion rates to the sensitive phenotype are also very high. Once the bacteria have become resistant, when the antibiotic is removed from the medium, a fraction as large as 95% of the population becomes susceptible again “almost immediately” according to Adam. et al [1], or in less than 100 generations according to George and Levy [3]. These results are incompatible with the hypothesis that genetic mutation is the sole cause of adaptive resistance, as otherwise i) the emergence of the resistant phenotype would be a sudden (or step wise discontinuous) event instead of appearing gradually [8]; and also ii) the resistant phenotype would not be easily reversible. For this to happen, back mutations would be required in the originally altered bases (or a compensatory mutation somewhere else), which is estimated to occur with an extremely low probability (10^−9^ or less) [12].

It has been suggested that a combination of epigenetic processes such as methylation and stochastic gene expression, may be driving the emergence of adaptive resistance [1, 5, 13]. Specifically, it has been proposed that DNA methylation by the DAM methylase could be responsible for: i) the presence and inheritance of different gene expression profiles [13–18] and ii) the variability in expression observed for methylated genes.

The activity of the DAM methylase gene has been found correlated with the emergence of adaptive resistance. In fact, high expression of the DAM methylase gene, increases the survival rate by a factor of five in cells treated with nalidixic acid [1]. Furthermore, this heightened resistance is consistent with a two fold increase in the expression of efflux pumps [1]. Therefore, DNA methylation affecting the activity of efflux pumps, could be a very likely explanation of the increased resistance observed experimentally, along with the fact that it will produce enough heterogeneity in the population for antibiotic selection to act on [1, 13, 15, 16].

In the light of these experimental observations, we propose a theoretical model to quantitatively determine what specific elements are essential for the emergence of reversible resistance. One key hypothesis of the model is that the Efflux Pump Regulatory Network (EPRN from now on) is a target of epigenetic modifications. Specifically, these modifications will produce variability in the gene expression patterns of the EPRN transcription factors. After cell division, such gene expression patterns will be inherited from mother to daughter and will consequently impose correlations in the dynamics of the EPRN across generations. Our results indicate that this mechanism is central for the emergence of adaptive resistance.

Our model also predicts that epigenetic variability and mother-daughter correlations are not sufficient to explain reversibility of resistance. Here, we demonstrate that reversibility is a consequence of a trade-off between the benefit of efflux pumps, keeping the antibiotic below lethal levels, and a cost associated with their activity, as they are known to pump out essential metabolites and therefore slow down cell growth [18].

Our theoretical framework aims at identifying and deciphering the role of each phenomenological observation in the emergence of adaptive resistance in order to provide a comprehensive and quantitative picture of this reversible phenomenon.

## Results

### Single Cell Efflux Pump Model.

**Fig 1:**
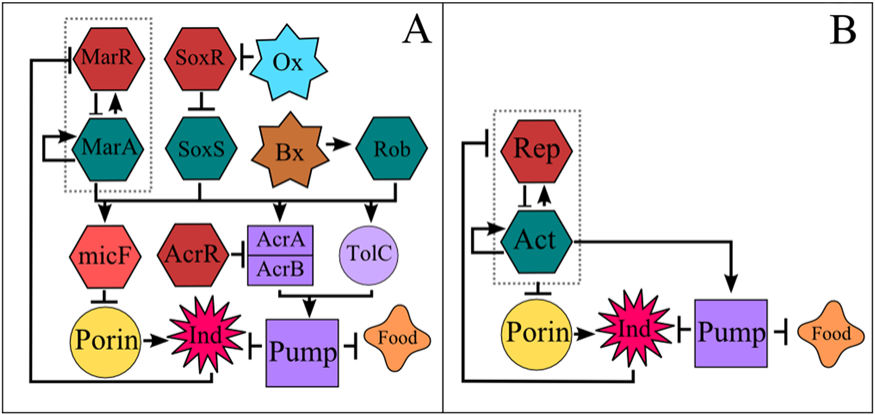
Efflux Pump Regulatory Network. Arrows indicate positive regulation. Blunt arrows indicate repression. A) Literature base reconstruction of the AcrAB-TolC efflux pump regulatory network of *Escherichia coli* as reported on [9]. B) Simplified version of the AcrAB-TolC efflux pump regulatory network (EPRN). The activator (Act) and repressor (Rep) are two Transcriptional Factors that belong to the same transcriptional unit (EPRN operon, indicated by the dashed line). When the repressor occupies its DNA binding site, the expression of the operon is restrained. Nonetheless, when the antibiotic (or inducer, *Ind*) enters the cell, it inactivates the repressor by binding to it, allowing the operon to be actively transcribed, promoting the production of pumps and decreasing the synthesis of porins (this last process is known to occur through an intermediary). Both food and inducer are expelled by the efflux pump system. In the population model, a reduction in food concentration implies an increase in the division time.

There are several EPRNs present in bacteria, and most of them share a number of prototypic characteristics that make them qualitatively similar to each other. We have constructed a single-cell model based on the acrAB-tolC system present in *Escherichia coli*, since it has been historically the most widely studied and well characterized system [19–28]. Fig. 1A shows a complete version of the acrAB-tolC regulatory network of *Escherichia coli* according to reference [9], whereas Fig. 1B shows a simplified version of it. This simplified version includes the main components of the acrAB-tolC system which are present not only in *E*. *coli* but also in other gram negative bacteria [9]. Note that the network depicted in Fig. 1B can be embedded into the larger network shown in Fig. 1A. For the reduced network we have chosen only the most dynamically important elements. This choice was not arbitrary but based on extensive numerical simulations that identified the essential components of the network, and eliminated the ones that did not provide additional information or present significant changes in the dynamics when removed (see S1 Text and S1 Fig.). For instance, we found that AcrR only reduces the expression of AcrA and AcrB linearly, and its role could be accounted for, by just changing the transcription rate of the AcrAB operon. This result is in agreement with previous research that indicates that AcrR only fine-tunes the expression of AcrAB to prevent unwanted expression [29], a scenario that becomes irrelevant when the antibiotic is introduced. All the results presented in this work were obtained with the simplified network. Nonetheless, the simplified network gives essentially the same results as the more complete version of this system (see S1 Fig.). Therefore, without loss of generality or biological realism, we use generic names such as *Activator*, *Repressor*, etc., to refer to the components of the network under study instead of *marA* or *marR*, etc. It is worth mentioning that the acrAB-tolC system responds to several antibiotics and also to non-lethal chemicals (such as salicylate), some of which were used in the adaptive resistance experiments mentioned earlier [20–22]. We will use the terms *antibiotic* and *inducer* indistinctly to refer to any chemical that promotes the activation of the EPRN.

The main idea behind the dynamics of the EPRN is the following. Two transcription factors are present: an activator and a repressor, both belonging to the same operon (hereafter referred to as the EPRN-operon). They auto-regulate their own transcription by binding to the EPRN-operon promoter region. In the absence of antibiotic the EPRN-operon is expressed at low levels [22, 23]. This is because the repressor molecule has a higher binding affinity (at least four times) than the activator [24, 25] and therefore the operon transcription is mainly restrained [26]. When the antibiotic enters the cell, usually via membrane porins, it binds tightly to the aforementioned repressor molecule, inactivating it. This new repressor-inducer conformation is now unable to bind to the EPRN-operon promoter region, allowing the activator to be transcribed. Since the activator promotes its own expression, it increases its concentration rapidly, boosting the production of efflux pumps; at the same time it represses the expression of membrane porins which reduces the intake of antibiotics [24, 27, 28].

We use a system of stochastic differential equations (see Supporting Information) to model the single-cell dynamics of the EPRN shown in Fig. 1B. Validation and parameter calibration for this system were done by comparing our simulations with experimental data from *E*. *coli* wild-type cells and ΔtolC mutant strains [30] (see S1 Text, S1 and S2 Tables and S2 and S3 Figs.). The efflux of antibiotics depends on two main parameters: the transcription rate β_0_ of the EPRN operon, which ultimately affects the amount of available pumps, and the pump efficiency ε_I_ that controls how much antibiotic the pumps can expel at a certain time. We will see later that by introducing cell-to-cell variability and mother-daughter correlations in these two parameters, highly resistant populations can arise.

Importantly, due to the large size of the parameter space, we do not perform an exhaustive parameter search to determine the complete regions over which our results hold. Rather, in S1 Table we present a set of parameters that qualitatively reproduce the experimental observations. Nonetheless, in the Supporting Information we provide a sampling of a wide region of the parameter space for which our results hold (see S1 Text and S4 and S5 Figs.).

### Population model: Variability and inheritance.

The population consists of a set of replicating cells, each one represented by a copy of the system of equations governing the dynamics of the EPRN (formally presented in the SI). Each cell runs internally its own system of equations independently from other cells. These cells will grow or die depending on their internal concentration of nutrients and antibiotics, respectively.

Cell-to-cell variability is implemented by slightly changing the two parameters that most affect the capability of the pumps to reduce the internal concentration of antibiotics. One such parameter is the efficiency ε_*I*_ of the efflux pumps. A high efficiency will correspond to a decreased toxicity of the antibiotic, which is due to pumps operating more rapidly or with greater specificity. We assume that changes in ε_*I*_ are caused by genetic mutations. However, since mutations alone cannot account for the rapid emergence of adaptive resistance [1, 3, 4, 6], we also implement variability in the transcription rate β_0_ of the EPRN operon. As stated in the introduction, we assume that cell-to-cell variations of this parameter are caused by epigenetic processes, most likely methylation [1, 14–17].

It is known that different methylation patterns can produce different transcription rates by changing the DNA binding affinity of some transcription factors [14–17]. We found 12 DAM methylation sites (GATC) in the regulatory operon of the AcrAB-TolC efflux pump system in *E*. *coli* (see SI). Each site can be in two states: either it is methylated or it is not. Therefore, there are 2^12^ = 4086 possible methylation patterns in this operon. As there are several possible patterns, we will assume that β_0_ (the transcription rate) changes as a continuous variable. However, this assumption is not crucial. In fact, even if only 1% of these 2^12^ possible methylation patterns were attainable and capable of producing different transcription rates, we would still have about 40 different patterns. We will see later that even under this scenario in which the values of β_0_ are discrete and finite, the same qualitative results are obtained. (See the section entitled “Emergence of the highly resistant phenotype” below.) Cells with different values of β_0_ will produce pumps at different rates, which in turn affect their survival. Variability in the population is then introduced by selecting, for each cell, the parameters β_0_ and ε_I_ from Gaussian distributions G(μ_β_, σ_β_) and G(μ_ε_, σ_ε_), respectively (in each case μ is the mean and σ^2^ is the variance). On the other hand, inheritance is implemented by correlating the mean values of these Gaussians across generations.

To illustrate the inheritance mechanism in our model, let us consider the *i* ^th^ cell at generation *t*, which has a transcription rate β_0_ (*i*, *t*). Then, the transcription rates β_0_(*i*_1_, *t* + 1) and β_0_(*i*_2_, *t* + 1) of its two daughter cells *i*_1_ and *i*_2_ at generation *t* + 1 will be drawn from the Gaussian G(β_0_(*i*, *t*), σ_β_), which has average μ_β_ = β_0_(*i*, *t*). In other words, at each generation and for each cell, β_0_ is drawn from a Gaussian distribution G (μ_β_, σ_β_) whose average μ_β_ is the value of β_0_ previously owned by the corresponding mother. This mechanism, which clearly correlates the parameters β_0_ along cell lineages, models the fact that methylation patterns that affect gene expression can be inherited with certain variability [14–17].

Inheritance in the pump efficiency ε_*I*_ is implemented in an analogous way, but using the corresponding Gaussian distribution G (μ_ε_, σ_ε_). However, since we are assuming that changes in β_0_ are epigenetic whereas those in ε_*I*_ are genetic, the time-scales at which significant modifications in these parameters occur, are very different. For it is known that phenotypic modifications due to epigenetic changes happen at rates at least one order of magnitude faster than those due to genetic changes [31]. Therefore, we have set σ_β_ = 20σ_ε_ in all our simulations, with σ_β_ = 0.1 and σ_ε_ = 0.005. Among the implications of using such small variances are that the changes in gene expression and pump efficiency between mother and daughter cells occur gradually. We also implemented for σ_β_ a uniform distribution between 0 and 10, which allows abrupt changes in gene expression between the mother and daughter cells. However, when this type of abrupt changes are allowed in σ_β_, adaptive resistance is not observed (see S1 Text and S6 Fig.). We do not know, based on experimental measurements, which of the two mechanisms mentioned above (i.e. uniform vs Gaussian randomness) is more compatible with the effect caused by methylation. But, as we will see in the next section, our model predicts that when Gaussian distributions with small variances are used, adaptive resistance emerges, which is not the case for uniform distributions (see S1 Text and S6 Fig.). Additionally, we also tested σ_β_ = Cσ_ε_ where 10 ≤ C ≤ 50 with no significant changes in the results.

In order to quantify the effect that each type of inheritance has on the emergence of the resistance phenotype, we implemented four different scenarios:

1. Control simulation: There is no inheritance, only variability. The distributions G (μ_β_, σ_β_) and G (μ_ε_, σ_ε_) remain the same for all the cells in the population and throughout generations.
2. Genetic inheritance: Mother-daughter correlations are implemented only in the pump efficiency ε_*I*_ but not in the transcription rate β_0_.
3. Epigenetic inheritance: Mother-daughter correlations are implemented only in the transcription rate β_0_ but not in the pump efficiency ε_*I*_.
4. Mixed inheritance: Mother-daughter correlations are implemented in both the transcription rate β_0_ and the pump efficiency ε_*I*_.

### Population model: Cell duplication and death.

The synthesis and functioning of efflux pumps are associated with an energetic cost that must be taken into account. First, the pumps are very unspecific on its substrates [21, 32, 33]. Thus, in addition to antibiotics, they expel metabolites necessary for cell growth and division [34]. For instance, the acrAB-tolC efflux pump, is known to recognize a broad spectrum of chemicals. It also has a biased affinity towards phenolic rings, which are not only constituents of inducers of the system such as salicylic acid, but also of amino acids such as tyrosine [32, 35]. Second, the synthesis of the pumps themselves (large protein complexes) and their functioning consume energy [9, 32, 35]. Therefore, it is reasonable to assume that the production and functioning of the pumps will slow down cell growth. This assumption is supported by experimental observations indicating that over-expression of efflux pumps is correlated with both high levels of resistance and decreased growth [18, 36, 37]. In our model we set this cost by making the internal concentration of nutrient in each cell depend inversely on the amount of pumps (see Eq. (6) in S1 Text). The net result is a slowdown of the cell division rate, because a minimum internal nutrient concentration is required for division to happen. Thus, when the internal concentration of nutrients reaches a certain threshold (θ_*F*_), the cell divides consuming the nutrient load, *F*. The two daughter cells start anew with a food load *F* = 0 and will have to accumulate resources again in order to divide. Clearly, the division time (the time it takes to reach the threshold θ_*F*_) depends on the amount of pumps, which in turn depends on the transcription rate β_0_ and the concentration of inducer (see S1 Text and S7 Fig.).

We have also included cell death in our population model. In order for the cell to survive, the efflux pumps need to keep the internal antibiotic concentration below the lethal level θ_I_. Whenever this threshold is reached, the cell dies and it is removed from the population.

### Emergence of the highly resistant phenotype.

**Fig 2:**
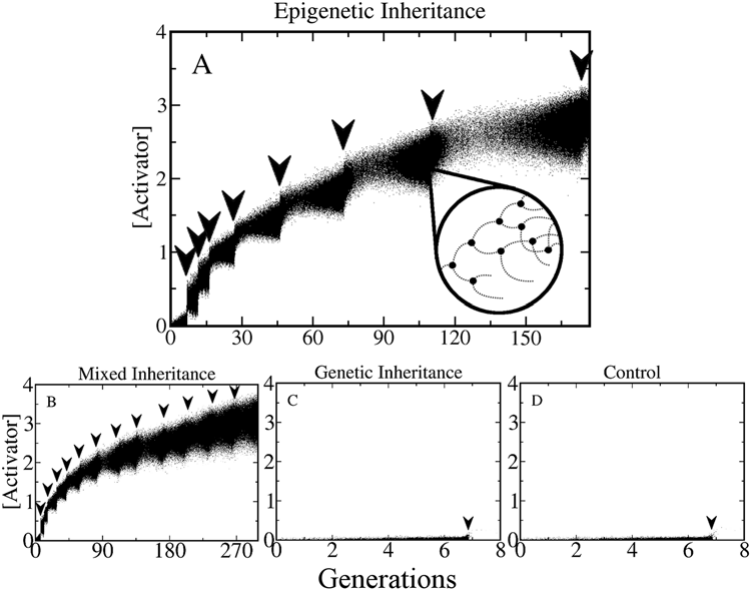
Emergence of the high resistance phenotype. This figure shows tracking plots of populations growing in successively increasing concentrations of antibiotic. For each cell the concentration of Activator is plotted at constant time intervals (dots). The arrows indicate the times at which antibiotic shocks were applied, which happened every time the population reached a maximum size of N = 5000. The four panels correspond to the four different inheritance scenarios mentioned in the main text. (A) Only epigenetic inheritance is implemented. (B) Mixed Inheritance. (C) Only genetic inheritance and (D) control (no inheritance). The inset in (A) shows a zoomed in representation of the tracking plot, where one cell lineage is followed as it goes through several cell divisions and deaths. Since dead cells are removed immediately from the population, their expression is no longer visible and their curves terminate abruptly (causing a step-like structure). Note that high levels of resistance can be achieved only when there is epigenetic inheritance (A and B). Otherwise, the entire population dies after the first shock (C and D). The unit of time corresponds to one cell cycle for β_0_ = 1 and with no antibiotic (see S6 Fig.).

Fig. 2 shows typical tracking plots of the activator concentration for the four scenarios mentioned above (control, genetic inheritance, etc.). As in the experiments reported in Refs. [1, 3, 6–8, 37], we subject the population to successive antibiotic shocks, each one gradually increasing the external concentration of antibiotic by an amount Δ*I*_*n*_. Thus, after *M* shocks the external antibiotic concentration will be $I_{ext}=\Sigma_{n=1}^{M}\Delta I_{n}$. After each antibiotic shock, indicated by downward arrows in Fig. 2, many cells die and only few cells survive. We let the few surviving cells grow and divide in the new antibiotic concentration until the population reaches a size N = 5000, at which point another antibiotic shock is applied (further increasing the external antibiotic concentration). As can be observed in Fig. 2, only when epigenetic inheritance is present the population is able to endure successive increments of antibiotic, reaching very high levels of resistance. By contrast when epigenetic inheritance is not present, every cell in the population dies after the first shock. The above results show that in our model variability alone is not enough for the emergence of adaptive resistance (Fig. 2D). Analogously, genetic inheritance, which essentially consists of mother-daughter correlations occurring at long time scales, cannot give rise by itself to adaptive resistance either (Fig. 2C).

For such adaptive resistance to emerge, short-term mother-daughter correlations in the transcription rate β_0_ of the EPRN operon need to be implemented in the model. Note that when both genetic and epigenetic inheritance are present (Fig. 2B) the population tolerates a higher number of antibiotic shocks in the same time interval than when only epigenetic inheritance is present. Fig. 2A also shows that the more resistant the cells become, the slower their division rates get. Indeed, the time it takes for the population to reach a size of N = 5000 in order to apply the next antibiotic shock, (indicated by the separation of the arrows), becomes longer as the population survives in higher antibiotic concentrations (this effect can also be seen in S7 Fig.). It is important to mention that if the antibiotic shocks occur at high frequencies (for instance less than 10 generations between two successive shocks), or if each antibiotic shock is much more intense (e.g. twice or more) than the previous one, we observe no surviving cells whatsoever in any of the four scenarios.

It is also worth noting that if a discrete distribution for β_0_ with 40 different values is used instead of a continuous Gaussian, the same qualitative results are obtained, as can be seen in S8 Fig. Therefore, adaptive resistance occurs even in the presence of moderate variability in gene expression, as long as there are mother-daughter correlations in such variability.

### Reversibility.

**Fig 3:**
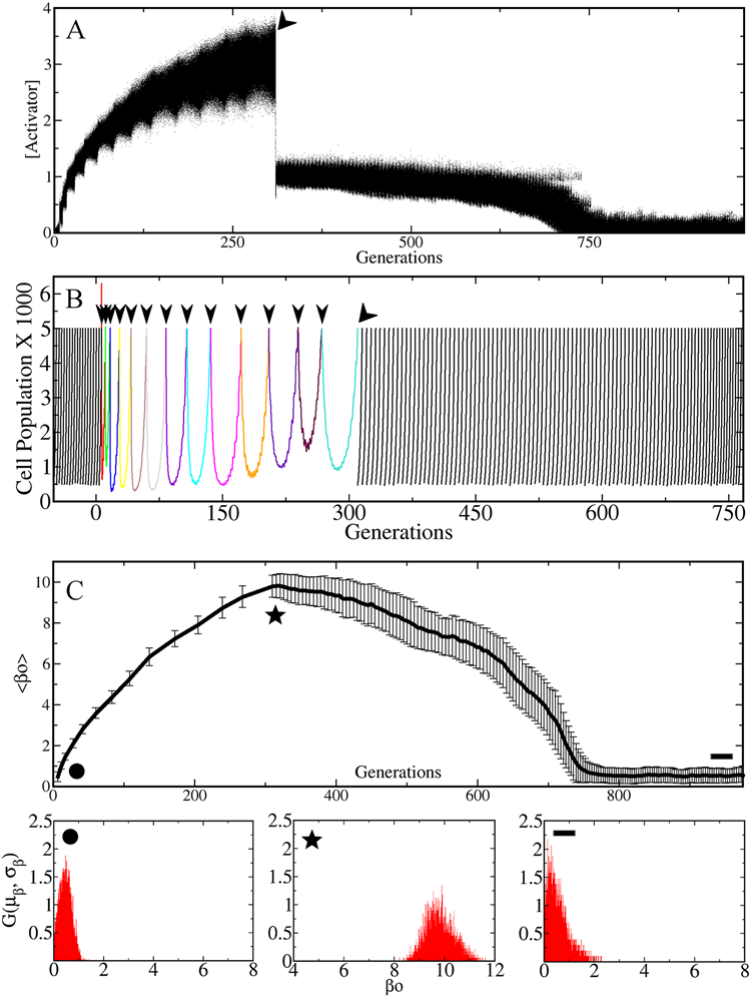
Reversibility of the resistance phenotype. (A) This tracking plot shows that the expression of the activator increases while the antibiotic shocks are applied as in Fig. 2. Then, when the antibiotic is removed (indicated by the tilted black arrow), the expression of the activator decreases abruptly and eventually reaches its basal level. (B) Size of the population as a function of time for the same simulation as in A. After each antibiotic shock (small black arrows) the population size decreases exponentially and the recovery time becomes longer with each shock. After the antibiotic is removed (tilted black arrow) the population comes back again to its wild-type (WT) growth rate. To carry out the simulations in the antibiotic-free phase, every time the population reached the maximum size N = 5000, we took a random sample of 10% of the cells and made them grow without antibiotic, until the population reached again this maximum size, and so on. (C) Average transcription rate μ_β_ = ⟨β_0_⟩ in the population as a function of time. Note that the average increases while the shocks are applied and then gradually comes back to small values when the antibiotic is removed. Error bars indicate the standard deviation. It can be observed that the standard deviation increases with the antibiotic stress. The panels below show the full distribution G (μ_β_, σ_β_) at three different times: before any antibiotic is introduced (circle); after several antibiotic shocks (star); after a long period of time without antibiotic (line). Time is measured generations, being one generation the time it takes for a cell with β_0_ = 1 to reach θ_F_ starting from F = 0.

So far, the difference between genetic and epigenetic inheritance consists on one hand, in the time scales at which these two processes produce phenotypic changes in the population, and on the other hand in the parameters they affect. Genetic inheritance affects the pump efficiency ε_*I*_ whereas epigenetic inheritance affects the transcription rate β_0_. Another important difference is that changes caused by genetic mutations are very unlikely to be reversible whereas epigenetic changes are much more likely to be reversible [31, 38]. To test if our model can reproduce the experimentally observed reversibility, we replicate the simulations described in the previous section (where levels of resistance are ramped higher) for the mixed scenario. But now, after several rounds of selection, we remove the external antibiotic and allow the cells to grow and divide without stress. (The antibiotic is removed by setting *I*_ext_ = 0 in Eq.(5) of the SI.) Fig. 3A shows that the concentration of the activator abruptly decreases when the antibiotic is removed (indicated by a big black arrow), eventually reaching its basal level of expression. Fig. 3B shows the size of the population as a function of time across the successive antibiotic shocks, and then in the free medium. The population size decreases exponentially each time an antibiotic shock is applied. However, after some time, the surviving cells grow and divide restoring the population size to the maximum size N = 5000. Note that when the antibiotic is removed the population growth returns to its wild-type behavior. These results are qualitatively similar to those observed experimentally [1, 3–8, 9]. However, this fact does not necessarily mean that the cells return to their wild type levels of susceptibility. For the cells that have reversed back to a sensitive phenotype could still have very high values of transcription rate, β_0_, and this high rate would imply that as soon as the antibiotic is applied again, the activator and the efflux pumps will be produced rapidly and at high concentrations. At this stage, most of the cells would be able to survive easily almost any antibiotic shock making the system non-reversible.

The only way for the population to truly reverse to its wild-type condition and become susceptible again is to return to their initial distribution G (μ_β_, σ_β_), which is centered at low values of β_0_. We expect this to happen because cells with a small β_0_ duplicate faster than cells with large β_0_ (see S1 Text and S7 Fig.). Since the values of β_0_ are correlated across generations, cells with faster division rates (low β_0_) will eventually dominate the population, shifting the distribution G (μ_β_, σ_β_) towards the low β_0_ region. Indeed, the population average μ_β_ = ⟨β_0_⟩ of the transcription rate as a function of time is reported in Fig. 3C. Note that μ_β_ increases as the antibiotic concentration is ramped higher. Then, when the external antibiotic is removed the average transcription rate across the population μ_β_ decreases gradually, reaching the same value as in the original wild-type population. The lower panels in Fig. 3C show the distribution G (μ_β_, σ_β_) at three different time points throughout the simulation. We start with a distribution centered at low values of β_0_ (μ_β_ ≈ 0.5). Then, after several rounds of selection the distribution G (μ_β_, σ_β_) is shifted to relatively high numbers (μ_β_ ≈ 10). But then again, when the antibiotic is removed, the distribution eventually returns to its initial configuration.

### Genetic Assimilation.

**Fig 4:**
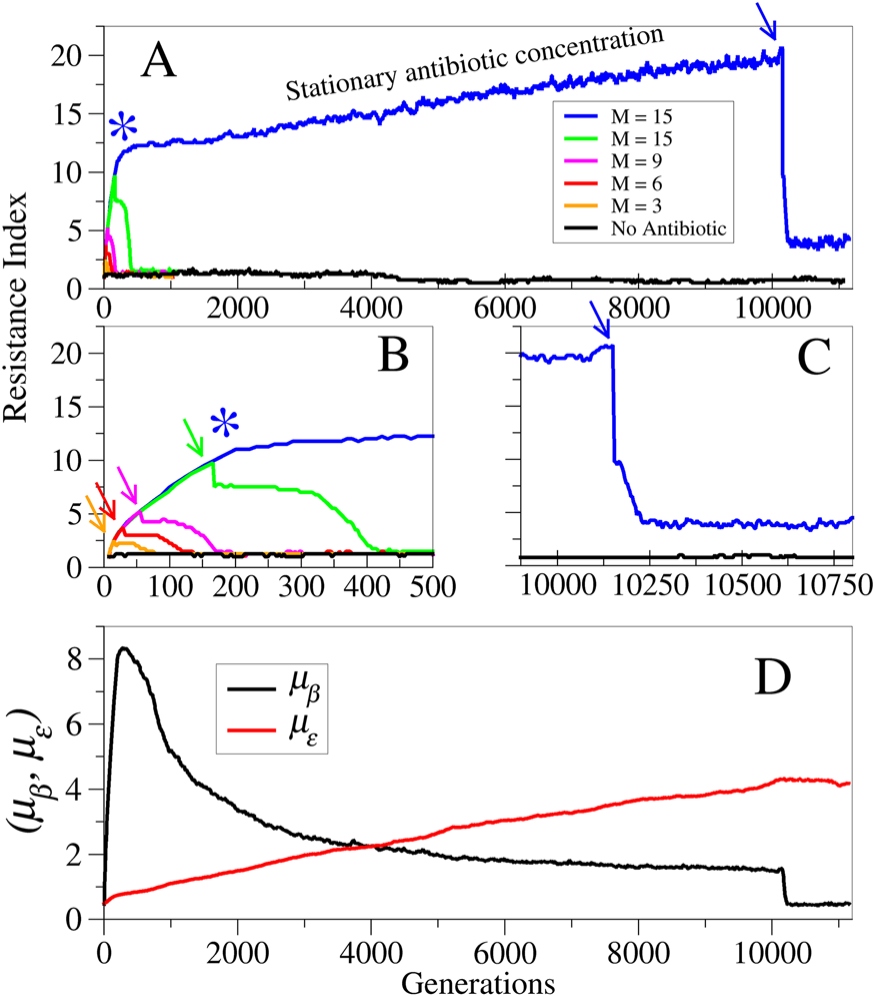
Genetic Assimilation occurs at longer time scales. (A) Resistance Index (RI) as a function of time for populations induced with *M* antibiotic shocks. The different curves correspond to different values of *M*, except by the black one which corresponds to a control population growing with no antibiotic. (B) Blow up showing the first 500 generations. For each curve, the corresponding arrow indicates the time at which the antibiotic is removed. In the case of the blue curve, the asterisk indicates the time at which the last antibiotic shock is applied, after which the antibiotic concentration is kept constant. (C) Blow up of the last part of the simulation showing the point at which the antibiotic is removed from the population corresponding to the blue curve. It can be observed that in this case the final stationary value of the RI is about five times higher than that of the control population. (D) Evolution of the average transcription rate μ_β_ and the average pump efficiency μ_ε_ for the population corresponding to the blue curve. Notice that as soon as the antibiotic concentration is kept constant, μ_β_ starts decreasing whereas μ_ε_ keeps rising until the antibiotic is completely removed. This shows that the evolutionary process does not reach a stationary state (or fixed point) in the presence of antibiotic.

In our model the time-scale to produce a phenotypic change due to genetic mutations is one order of magnitude larger than that needed to produce a phenotypic change due to epigenetic modifications. Thus, to observe any significant increase in resistance produced by changes in the pump efficiency we need to run the simulation for a longer time. Interestingly, by doing this we obtain a nonreversible resistance, first driven by our mechanism of epigenetic inheritance (which is reversible), and then fixed by genetic variation and inheritance of the pump efficiency. To observe this phenomenon, which can be considered analogous to genetic assimilation [39, 40, 41], we performed numerical experiments similar to the ones presented in the previous sections, where the population is first induced with *M* antibiotic shocks. The difference now is that we will let the population be in contact with the antibiotic for a very long time before removing it. To measure the level of resistance of the population throughout this process we define the Resistance Index (RI) as the maximum concentration of antibiotic that the population can endure with at least 10% of survival. (A similar measure was used in [3].) Fig. 4 reports the evolution of the RI for different populations subjected to a different number *M* of antibiotic shocks. In each case, the arrows indicate the time at which the antibiotic is removed. The results depicted in Fig. 4 show that the final stationary value of the resistance index (the one reached when the antibiotic is removed) depends on how long the population remains in contact with the antibiotic. The blue curve deserves special attention. In this case, *M* = 15 antibiotic shocks were applied, with the last shock occurring at the time indicated by the blue star. After this, the antibiotic concentration was kept constant until the time indicated by the blue arrow, at which the antibiotic was removed. Note that the RI keeps increasing even during the interval of steady antibiotic concentration. Note also that the final RI stationary value reached after the antibiotic is removed is five times larger for the blue curve than for all the other curves. It is worth noting that the black curve, corresponding to a control population growing in the absence of antibiotic, remains close to the initial low basal level throughout the entire simulation. Therefore, in our model antibiotic resistance occurs only as a response to the selective pressure imposed by the antibiotic and not by random genetic drift.

It is important to mention that the increase in the basal level of the RI shown in Fig. 4A when the population is kept in a high antibiotic concentration for a long time is non reversible. Indeed, Fig. 4D shows the evolution across generations of the average pump efficiency μ_ε_ and the average transcription rate μ_β_ for the population corresponding to the blue curve of Fig. 4A. From Fig. 4D it is apparent that μ_β_ increases only when antibiotic shocks are applied, namely, when the antibiotic concentration in the environment also increases. However, as soon as the antibiotic concentration is kept constant, even at a high value, the average transcription rate μ_β_ starts decreasing and reaches its initial low value at the end of the simulation. Contrary to this, the average pump efficiency μ_ε_ keeps rising as long as there is antibiotic in the environment, reaching a steady value only when the antibiotic is removed. Thus, exposing the population to a high antibiotic concentration for a long time produces a non-reversible shift in the pump efficiency distribution P(ε), permanently increasing the level of resistance of the population.

It is also important to emphasize the difference between the survival rate (SR) and the resistance index RI. The former is defined as the fraction of cells that survive an induction, and this fraction ranges from 0 (if no cell survives) to 1 (if all cells survive). On the other hand, the RI is the value of the antibiotic concentration at which the SR is 0.1 (i.e., the concentration of the antibiotic at which only 10% of the cells survive). Therefore, the RI does not have to be between 0 and 1. Actually, its value depends on the units used to measure the antibiotic concentration (in our case we use arbitrary units) and the capability of the population to resist the antibiotic. This capability, in turn, depends on the way β_0_ and ε_*I*_ are distributed across the population. In each cell, these parameters determine the fixed points of the system (only one fixed point exists for a given combination of β_0_ and ε_*I*_ in the range of concentrations explored in this work, see S1 Text and S9 Fig.). The results presented in Fig. 4 indicate that the permanent change in the RI observed after a long induction time is not due to transitions between fixed points, but to the fact that the unique fixed point of each cell moves throughout the evolution of the population.

## Discussion

Adaptive resistance in bacteria is observed after subjecting a population to gradual increments of antibiotic concentration. Regardless of the level of resistance reached through this process, (which can be very high), the resistance disappears after a few generations in the absence of antibiotic. Previous studies have independently identified epigenetic inheritance and phenotypic heterogeneity as important components involved in the emergence of adaptive resistance [1, 3, 4, 6, 7, 8, 11], but their role has never been evaluated quantitatively. Additionally, the molecular origin of reversibility observed in adaptive resistance has remained unclear.

In this study we present a theoretical framework that identifies the essential mechanisms for the emergence, evolution and reversibility of adaptive resistance. We constructed a single-cell dynamic model of a prototypic efflux pump regulatory network (EPRN) that incorporates the most updated information available in the literature. We calibrated this model with experimental observations for wild type and mutant *E*. *coli* strains. We then grew a population of such single cells with growth dynamics obeying simple rules such as division, death, variability and inheritance of gene expression patterns. For each cell in the population we compute their EPRN temporal dynamics. Through this model we demonstrate that heterogeneity and mother-daughter correlations affecting transcription rates, specifically those of the EPRN main regulators, can explain the gradual amplification of the multidrug resistant phenotype. By contrast, mother-daughter correlations implemented in the pump efficiency, and developing at longer timescales, were not sufficient to make the population adapt and survive to successive antibiotic shocks (but had a role in fixing resistance when the population had contact with antibiotics for a very long time). We also found that introducing a cost associated with the functioning of the EPRN was enough to explain the observed reversibility to the susceptible (non-resistant) phenotype.

A previous report [11] proposed that adaptive resistance developed as a consequence of heterogeneity in gene expression because cells that randomly have a high production of efflux pumps survive, and those that did not, die. Through our model, we were able to show that although heterogeneity in gene expression is necessary, it is not sufficient to explain the emergence of resistance, nor its gradual response, as epigenetic inheritance of gene expression patterns is also necessary. Epigenetic modifications can change gene expression patterns at short time scales, providing a mechanism by which cells can adapt to changing environments quickly. At the same time, it allows for enough flexibility: if the environment returns to its earlier state, a population whose fitness is compromised by the new gene expression patterns can return to its previous state in a short time.

Based on several experimental observations, another report [1] suggested that DNA methylation is a plausible mechanism driving this epigenetic inheritance. Methylation can indeed produce both the heterogeneity and epigenetic inheritance of gene expression patterns required for adaptive resistance to occur. Our results support this idea and specifically identify the regulatory regions of the main regulators of the EPRN as the most probable targets for the methylation process, as amplification of the antibiotic resistance do not occur without the mother-daughter correlations in gene transcription rates (see Fig. 2). Consequently, the process of DNA methylation in bacteria is potentially an important target for the development of therapeutic treatments in preventing the emergence of adaptive resistance.

It is important to stress that variability in gene expression is known to be essential for adaptive resistance to occur [11]. So, our model incorporates this variability with the additional feature that it has to be inherited. Whether or not this variability is caused by methylation is not the central point. Nonetheless we propose DNA methylation of the marRAB operon as the possible cause of this variability because: (i) it can be inherited; (ii) mutant cells in which methylation is lacking are much more susceptible to antibiotics [1]; (iii) it provides the necessary variability in short periods of time required for adaptive resistance to emerge [1]. However, regardless of the precise mechanism behind this variability, the important point in our model is the existence of inheritable variability that can be quickly developed. For our results show that some heritable mechanism modifying the transcription rates of an efflux pump regulatory network must be present in order to observe adaptive resistance.

Another interesting observation is the emergence of a stable form of resistance when the population is left in a medium with high concentrations of antibiotic for very long times. In our model this non-reversible resistance is produced by changes effectively improving the pump efficiency ε_*I*,_ meaning that the pumps become better at distinguishing antibiotics from nutrients, so that they can pump out the former at a higher rate than the latter. Although these genetic modifications are rare and insufficient to save the population initially, they become important at longer times, transforming into an alternate source of resistance without adverse effects. Therefore, this heritable trait will, at longer time scales, permanently increase the basal levels of resistance of the population when it is under selective pressure (see Fig. 4A). In our model genetic changes at each generation are small and increase the pump efficiency gradually (Fig. 4D). In reality, genetic changes, although rare, may produce abrupt changes in the level of resistance of the population. The important point is that the fast epigenetic changes occurring in the transcription rate allow the population to survive long enough as to develop more stable and efficient forms of resistance. This behavior is consistent with experimental observations, showing that bacterial populations that have been continuously exposed to antibiotics are permanently much more resistant than populations that have been not [1].

We have based our simulations on the regulatory scheme of the widely known acrAB-tolC efflux pump system, for which many of the kinetic parameters are still unknown. In our study, we aimed to identify the essential mechanisms that could explain and reproduce adaptive resistance and our results hold in a significant region of the parameter space and not only for the particular values presented in S1 Table (see S4 and S5 Figs.). However, performing a deep search in the parameter space of the equations could reveal important constraints; such as the timescales at which epigenetic inheritance or genetic mutations must occur; or even the amount of pumps that the cell needs to produce (which is to our current knowledge an unknown variable). We also explored alternative mechanisms that could yield resistance, such as uneven pump distribution in each cell division (one daughter cells takes the majority of the pumps) and increased mutation rates (which increases the variability σ_ε_ in the efficiency of the efflux pumps). The results, presented in the SI (see S1 Text, S10 and S11 Figs.) show that neither of these two mechanisms is able to produce adaptive resistance.

Our model provides an explanation for the emergence of adaptive resistance based on the cost and benefit of the biological characteristics of an efflux pump system. It does not only predict the behavior of populations subjected to different antibiotic shocks and at different time, but also a number of different phenomena observed experimentally in bacterial populations, such as phenotypic reversibility, genetic assimilation, and even the survival rates of populations that have been pre-induced with non-lethal antibiotic concentrations (see S1 Text and S12 Fig.).

## Supporting Information

S1 Fig Comparison between the complete vs the simplified EPRN models. Expression of the nodes corresponding to the activator (MarA in the complete network and Activator in the simplified network) and the pumps (AcrAB-TolC in the complete network and Pumps in the simplified network). Two external inducer concentrations are presented (I = 0 black curves, and I = 7 red curves) as well as two different values of the transcription rate β_0_, (β_0_ = 0 and β_0_ = 5). It can be observed that the curves for the complete and simplified networks are extremely similar in all cases.
(TIF)

S2 Fig Dynamical behavior of the SC-EPRN. We report the ratio Г_X_ = X_a_/X_w_ of expression levels of the network element X with antibiotic (X_a_) and without antibiotic (X_w_). Black bars indicate an increase in concentration in the antibiotic medium (Г_x_ > 1) whereas red bars indicate lower expression when the antibiotic is present (Г_x_ < 1). It can be observed that the presence of antibiotic triggers an overexpression of the activator operon (A and R), a reduction of the porins (Q), an increase in the production of pumps (P), and a reduction in the active form of the repressor (R*), as it has been reported in [1].
(TIF)

S3 Fig Behavior of the SC-EPRN for wild-type and mutant strains. The plot shows the concentration of the activator A as a function of time for wild type (P^+^, black circles) and pump deficient (P^−^, red squares) strains. Approximately, a twofold increase in the concentration of the activator in the mutant versus the wild type strains is observed in our simulations, which correspond to the experiments reported in [2]. In fact, this twofold increase was used to calibrate some of the parameters in the numerical simulation.
(TIF)

S4 Fig Parameters producing equivalent dynamics. By moving the values of the degradation rates γ_A_ and γ_R_ of the activator and the repressor, respectively, along the curve, we obtain the same qualitative results for the induction experiments as the one shown in Fig. 2 of the main text. The triangles show the particular values used to generate the plots in S4 Fig. The numbers between parentheses indicate the average increase of the antibiotic between two successive shocks. These results suggest that the conclusions of our model hold for a wide region in the parameter space and not just for the one particular point reported in S1 Table.
(TIF)

S5 Fig Adaptive resistance for equivalent parameters. Tracking plots for the activator corresponding to the 1^st^ (A), 2^nd^ (B), 3^rd^ (C) and 5^th^ (D) points in S3 Fig. Note that these plots are qualitatively similar to the one shown in the main text (Fig. 2), even though the plots here were obtain with different parameter values changing in almost one order of magnitude.
(TIF)

S6 Fig Uniform distribution for β_0_ with no correlations. This plot shows the size of the population as a function of time for the case in which the value of β_0_ for each cell in the population and for each generation is taken randomly with uniform probability from the interval [0, 10]. The upper arrow indicates the time at which the first antibiotic shock is applied, whereas the lower arrow indicates the application of the second antibiotic shock. Note that in this case in which there is no mother-daughter correlation in the value of β_0_, the population is not able to survive the second antibiotic induction, even though there is a relatively high variability in the population.
(TIF)

S7 Fig Division time as a function of the transcription rate β_0_ for different antibiotic concentrations. Each point is the average division time over 1000 cell division events (The average is necessary because of the presence of noise.) The black curve corresponds to cells growing in an antibiotic-free environment, while the red and green curves correspond to cells growing in antibiotic concentrations [I_ext_] = 1 and [I_ext_] = 3, respectively. Note that the division time increases with both the concentration of external inducer I_ext_ and the transcription rate (β_0_).
(TIF)

S8 Fig Discrete distribution for β_0_. Tracking plot for the activator in the case in which β_0_ takes 40 different discrete values (which represent about 1% of the theoretically possible 2^12^ different methylation patterns). In this case, in each replication the daughter cells can either acquire the same value of β_0_ than the mother with probability 0.5, of any of the two adjacent values with the same probability 0.25. The inset shows the distribution P(β) at the beginning of the simulation (blue histogram) and after several antibiotic shocks (red histogram). Note that the behavior of the system is essentially the same as in Fig. 2 of the main text, which indicates that a large number of methylation patterns is not necessary to obtain adaptive antibiotic resistance.
(TIF)

S9 Fig Fixed Points of the EPRN. A) Fixed point of the activator as a function of β_0_ for a fixed concentration of external inducer. B)-D) Stream plots on the Activator-Repressor plane for different values of β_0_ showing that there is only one fixed point in the range of parameters explored in this work.
(TIF)

S10 Fig Changing the epigenetic/genetic variability rate. (A) Increased mutation rates. Population size for a mixed model where the genetic variability was made equal to that of the epigenetic variability, σ_ε_ = σ_β_ = 1. We can observe that the population can endure a lot more antibiotic shocks (occurring at each peak) than the mixed model where the variance in the pump efficiency was much lower 20σ_ε_ = σ_β_ (see Fig. 3B). Also, cell death is significantly reduced (approx 15% after the first induction), which is at odds with the behavior observed experimentally, where cell death is much higher [4, 5]. (B) Inverted time-scales. Population size for a mixed model where the genetic variability was interchanged with the epigenetic variability; σ_ε_ = 0.1 and σ_β_ = 0.005. We can observe that the population dies immediately after the first antibiotic shock, which supports the idea that mutations alone cannot explain adaptive resistance. Time is measured in generations.
(TIF)

S11 Fig Uneven pump segregation and persistent cells. Plot of the population size as a function of time when random pump segregation is implemented without genetic or epigenetic inheritance. Each antibiotic shock is indicated by a change in color and by an arrow. Note that after the first shock just a few cells survive. These surviving cells are highly resistant because they can survive further antibiotic shocks. However, these cells cannot divide (the population size remains constant). This behavior is similar to the one observed experimentally in persistent cells [6].
(TIF)

S12 Fig Pre-induction increases survival rates. Survival ratio SR as a function of the antibiotic shock concentration I_ext_ for different pre-induction levels: I_pre_ = 0 (i.e. no pre-induction, black curve), I_pre_ = 0.25 (red curve), and I_pre_ = 0.5 (green curve). Note that the survival ratio increases with the pre-induction concentration I_pre_.
(TIF)

S1 Table Parameters for the single cell and population models. Values of the parameters that were used in the numerical simulations of both the single-cell and population models. The second column shows the values used to obtain the results presented in the main text, while the third column shows the values used for the alternative scenarios (presented in the SI). In the latter case the values that are different from those in the original model are shaded in gray.
(DOC)

S2 Table Meaning and interpretation of parameters. This table lists all the parameters of the model and gives their biological interpretation.
(DOC)

S3 Table Parameters for the complete network. Values of the parameters used for the numerical simulations of the complete Mar system depicted in Fig. 1A of the main text.
(DOC)

S1 Text Supporting Information for the main text and supplementary figures. (DOC)
