# Supplementary figures and images for: Adaptive Resistance in Bacteria Requires Epigenetic Inheritance, Genetic Noise, and Cost of Efflux Pumps
Source: PLoS One. 2015 Mar 17;10(3):e0118464. doi: 10.1371/journal.pone.0118464 (PMC4363326; doi:10.1371/journal.pone.0118464)

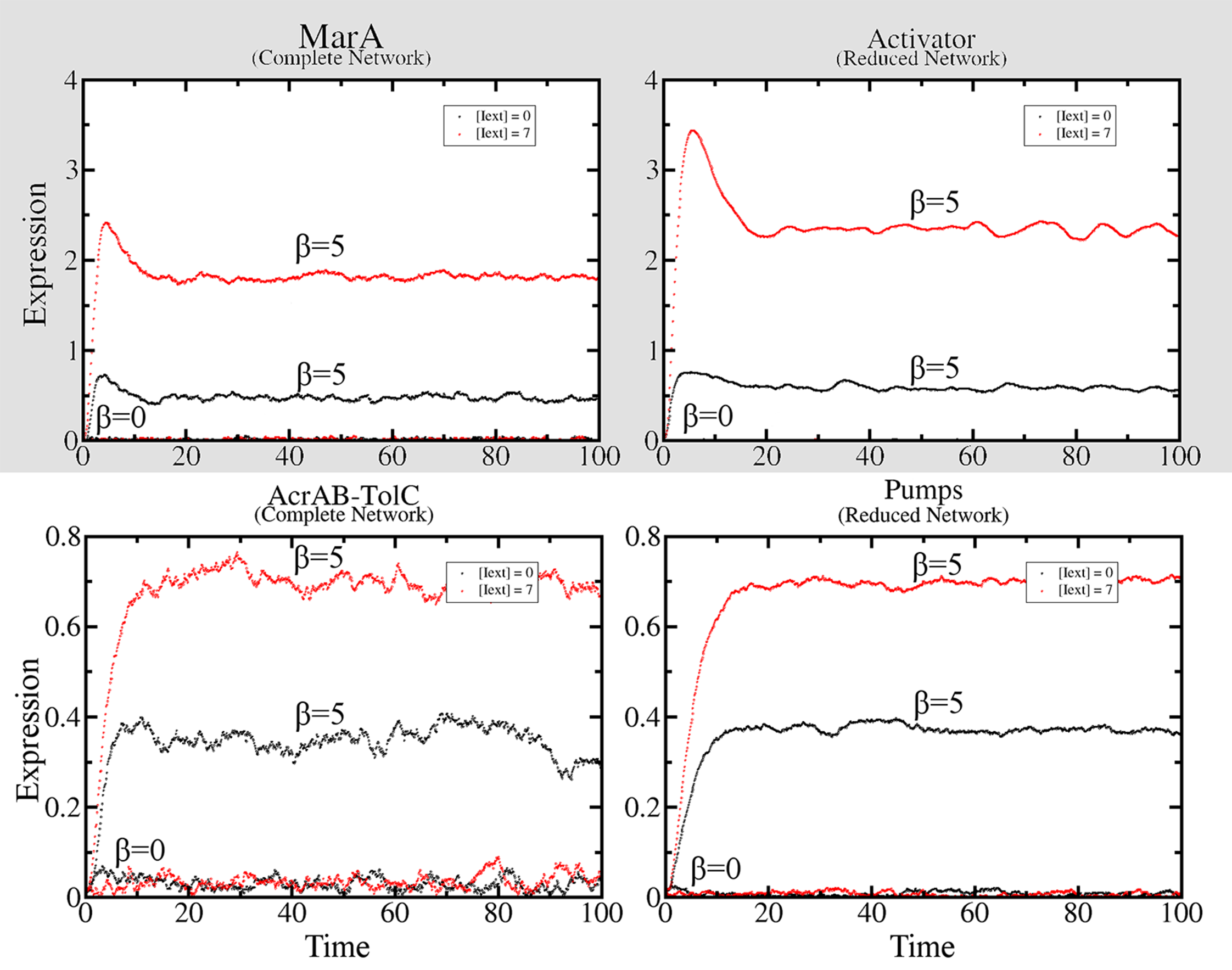

Supplement: S1 Fig — Expression of the nodes corresponding to the activator (MarA in the complete network and Activator in the simplified network) and the pumps (AcrAB-TolC in the complete network and Pumps in the simplified network). Two external inducer concentrations are presented (I = 0 black curves, and I = 7 red curves) as well as two different values of the transcription rate β0, (β0 = 0 and β0 = 5). It can be observed that the curves for the complete and simplified networks are extremely similar in all cases. (TIF) [file pone.0118464.s001.tif]

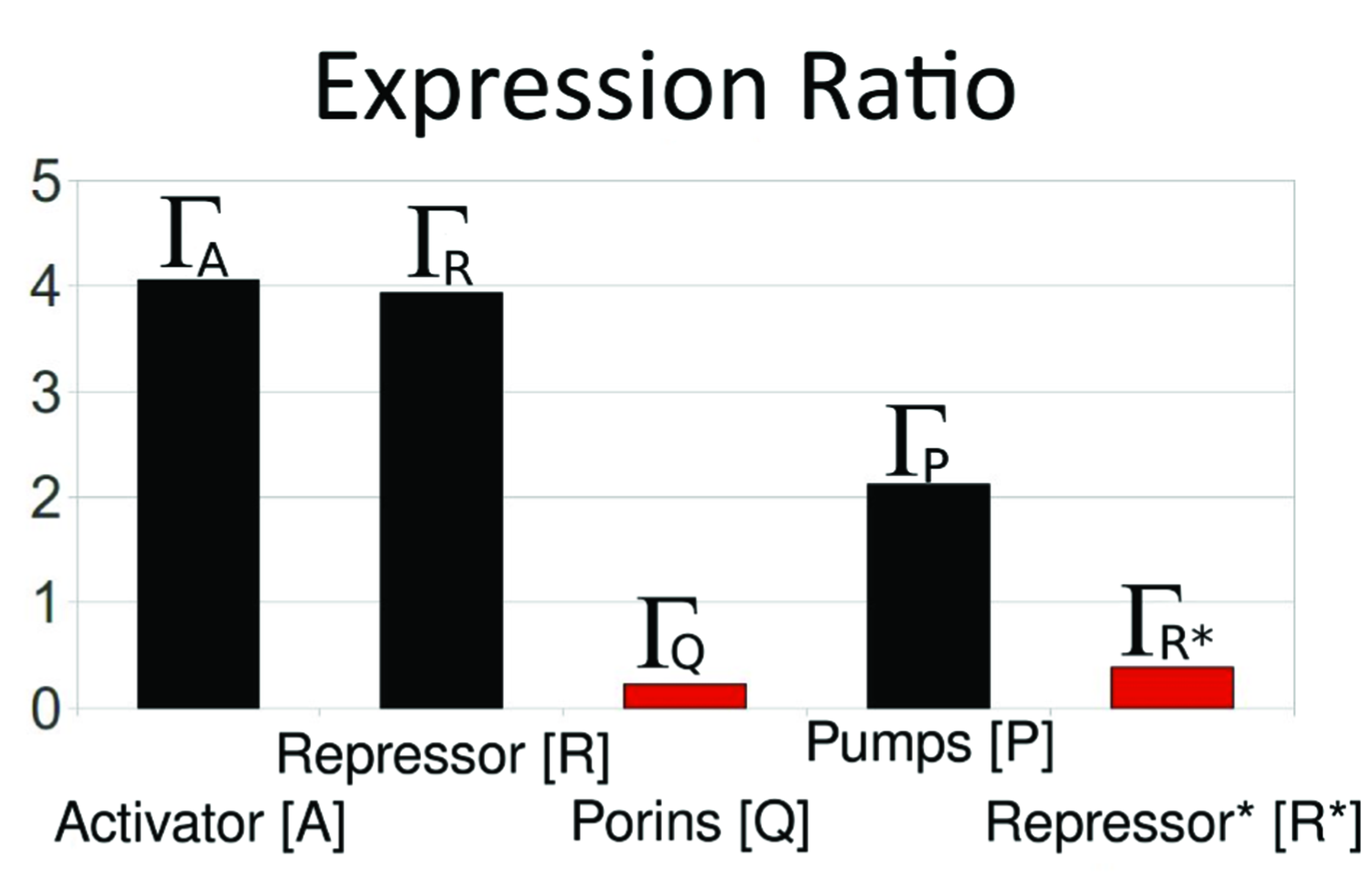

Supplement: S2 Fig — We report the ratio ГX = Xa/Xw of expression levels of the network element X with antibiotic (Xa) and without antibiotic (Xw). Black bars indicate an increase in concentration in the antibiotic medium (Гx > 1) whereas red bars indicate lower expression when the antibiotic is present (Гx < 1). It can be observed that the presence of antibiotic triggers an overexpression of the activator operon (A and R), a reduction of the porins (Q), an increase in the production of pumps (P), and a reduction in the active form of the repressor (R*), as it has been reported in [1]. (TIF) [file pone.0118464.s002.tif]

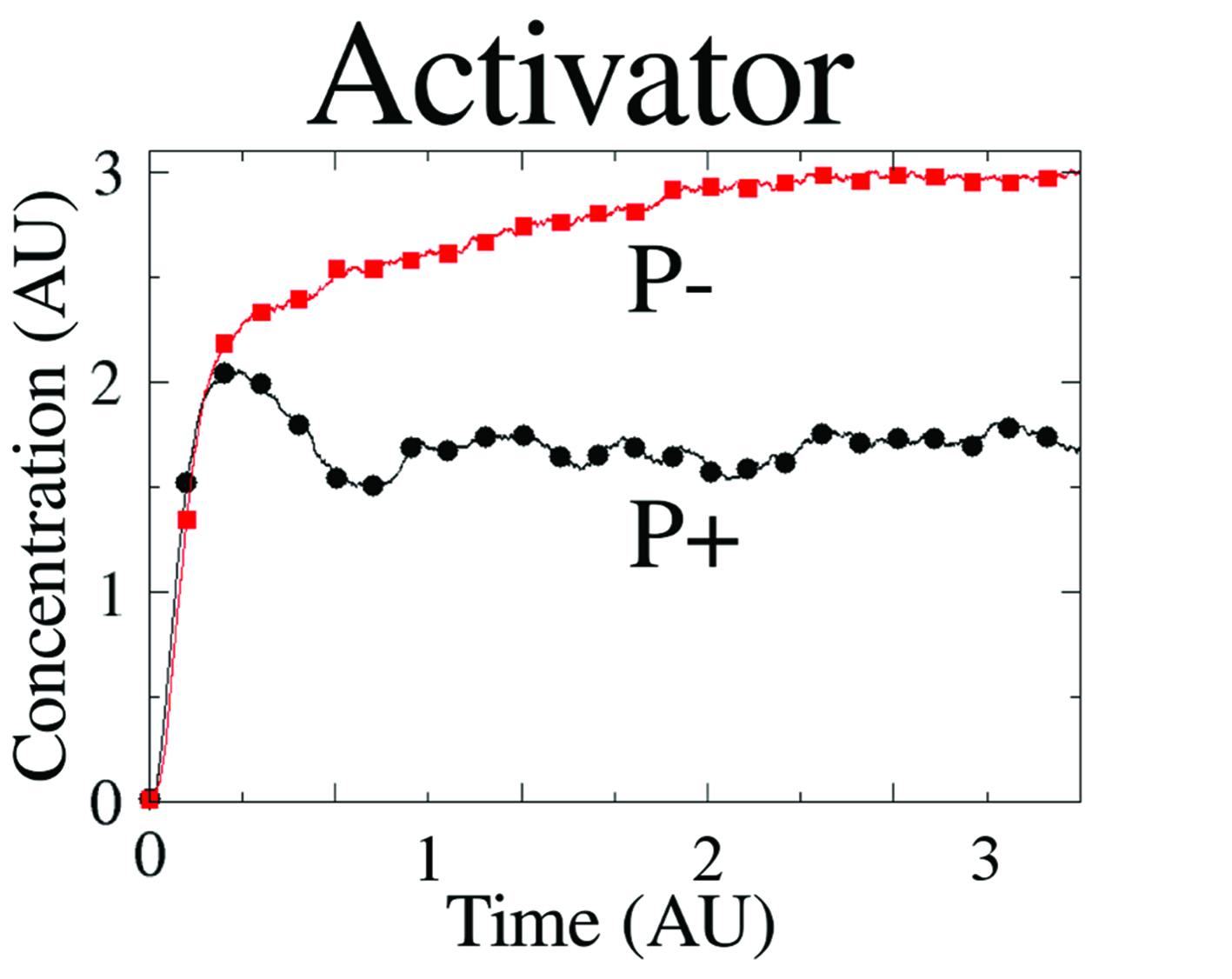

Supplement: S3 Fig — The plot shows the concentration of the activator A as a function of time for wild type (P+, black circles) and pump deficient (P−, red squares) strains. Approximately, a twofold increase in the concentration of the activator in the mutant versus the wild type strains is observed in our simulations, which correspond to the experiments reported in [2]. In fact, this twofold increase was used to calibrate some of the parameters in the numerical simulation. (TIF) [file pone.0118464.s003.tif]

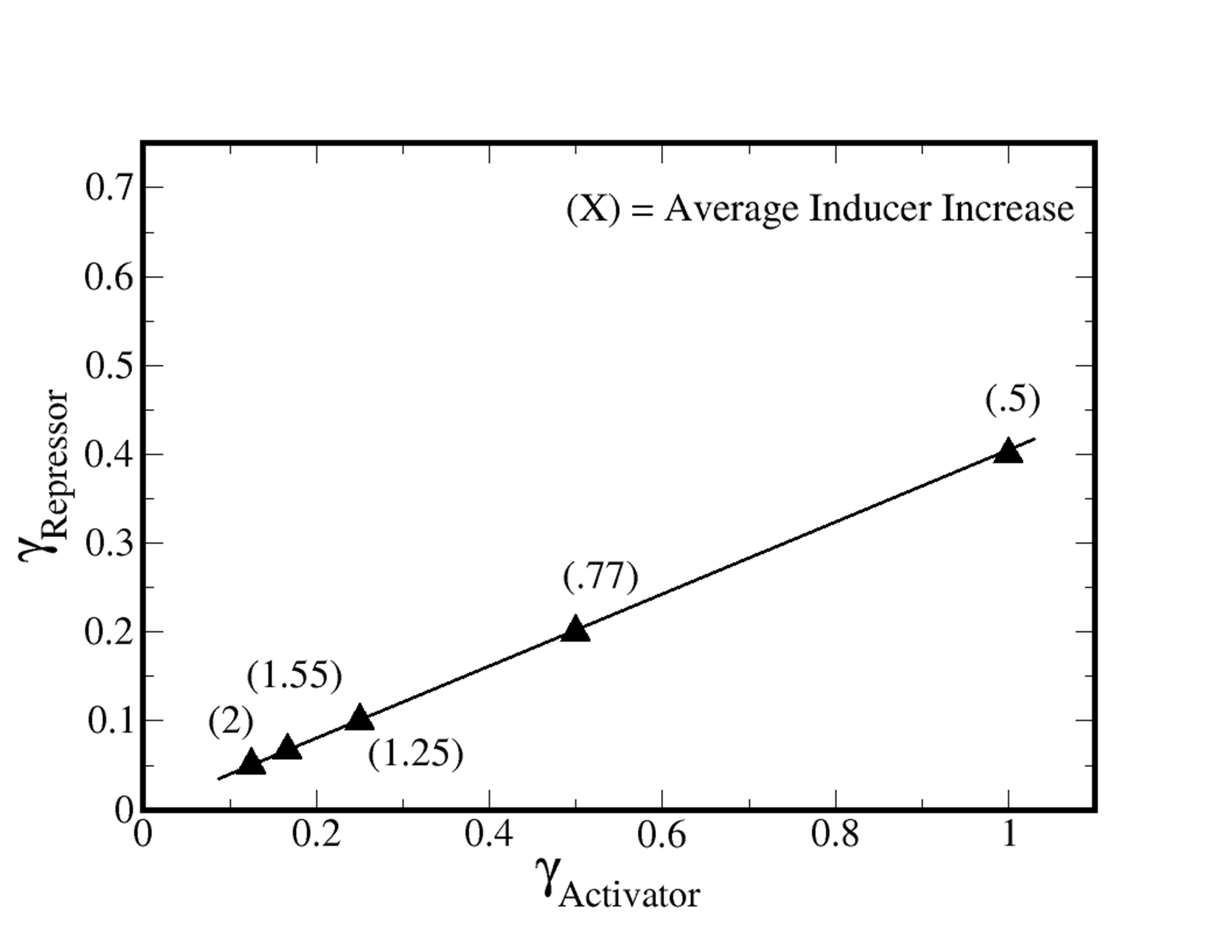

Supplement: S4 Fig — By moving the values of the degradation rates γA and γR of the activator and the repressor, respectively, along the curve, we obtain the same qualitative results for the induction experiments as the one shown in Fig. 2 of the main text. The triangles show the particular values used to generate the plots in S4 Fig. The numbers between parentheses indicate the average increase of the antibiotic between two successive shocks. These results suggest that the conclusions of our model hold for a wide region in the parameter space and not just for the one particular point reported in S1 Table. (TIF) [file pone.0118464.s004.tif]

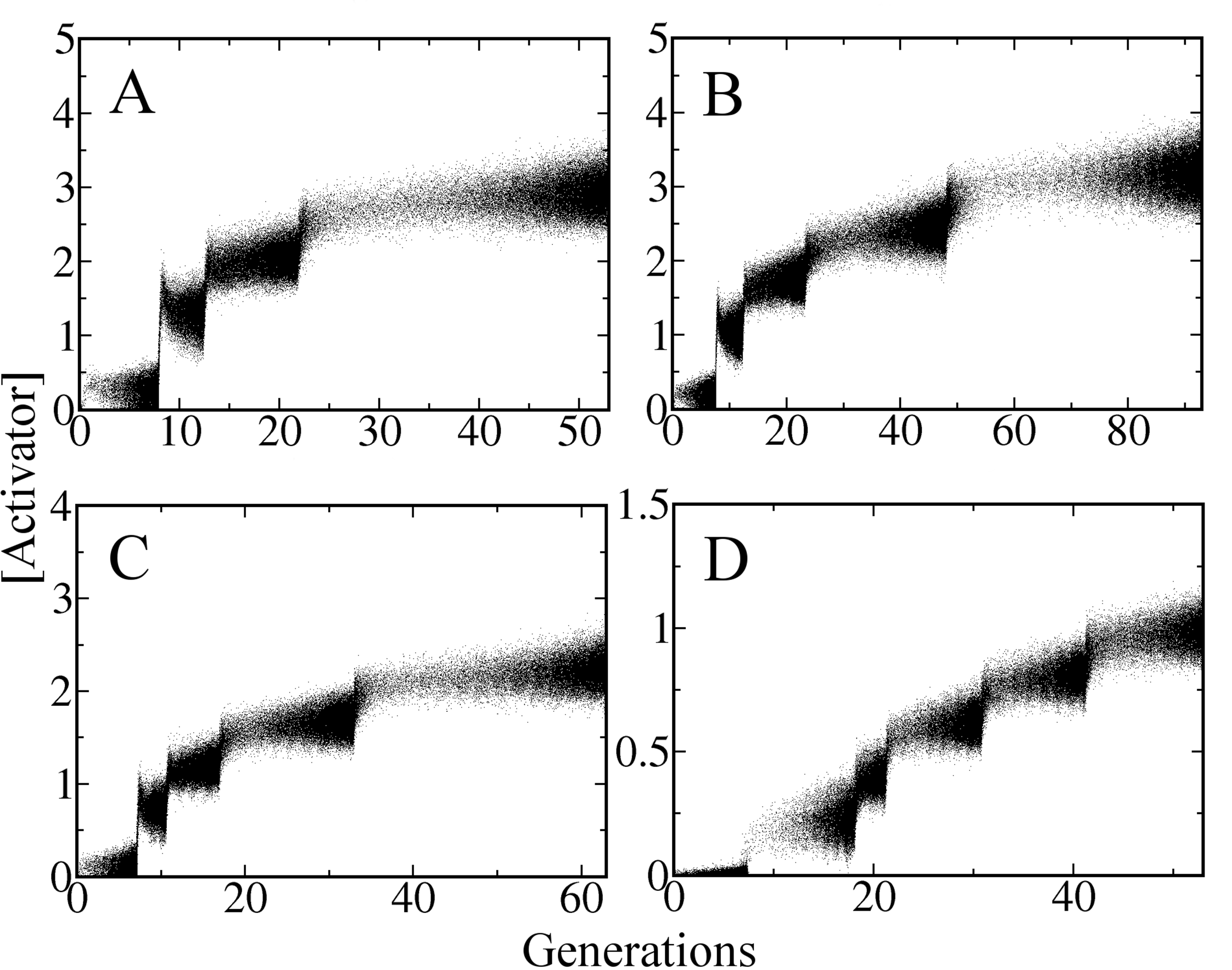

Supplement: S5 Fig — Tracking plots for the activator corresponding to the 1st (A), 2nd (B), 3rd (C) and 5th (D) points in S3 Fig. Note that these plots are qualitatively similar to the one shown in the main text (Fig. 2), even though the plots here were obtain with different parameter values changing in almost one order of magnitude. (TIF) [file pone.0118464.s005.tif]

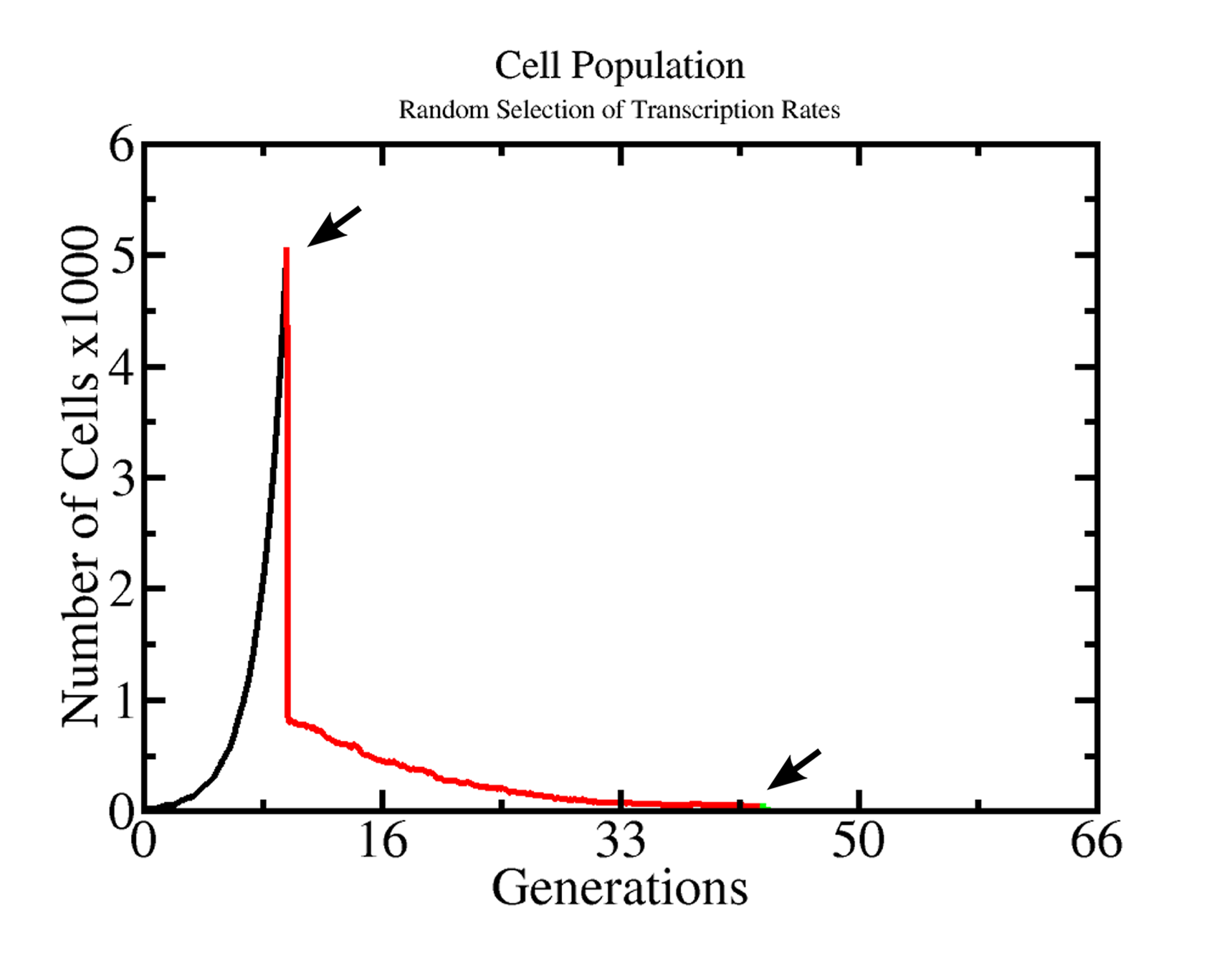

Supplement: S6 Fig — This plot shows the size of the population as a function of time for the case in which the value of β0 for each cell in the population and for each generation is taken randomly with uniform probability from the interval [0, 10]. The upper arrow indicates the time at which the first antibiotic shock is applied, whereas the lower arrow indicates the application of the second antibiotic shock. Note that in this case in which there is no mother-daughter correlation in the value of β0, the population is not able to survive the second antibiotic induction, even though there is a relatively high variability in the population. (TIF) [file pone.0118464.s006.tif]

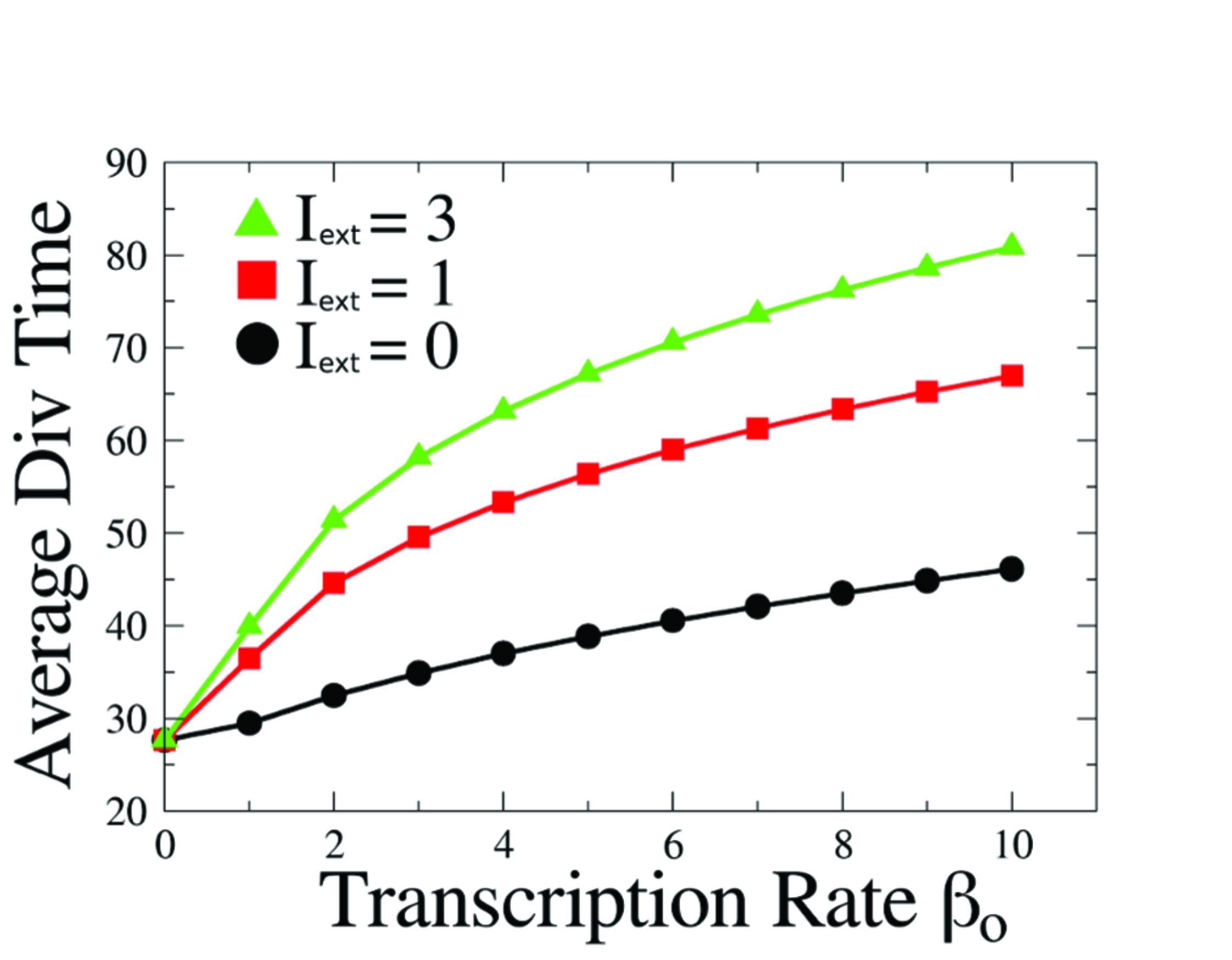

Supplement: S7 Fig — Each point is the average division time over 1000 cell division events (The average is necessary because of the presence of noise.) The black curve corresponds to cells growing in an antibiotic-free environment, while the red and green curves correspond to cells growing in antibiotic concentrations [Iext] = 1 and [Iext] = 3, respectively. Note that the division time increases with both the concentration of external inducer Iext and the transcription rate (β0). (TIF) [file pone.0118464.s007.tif]

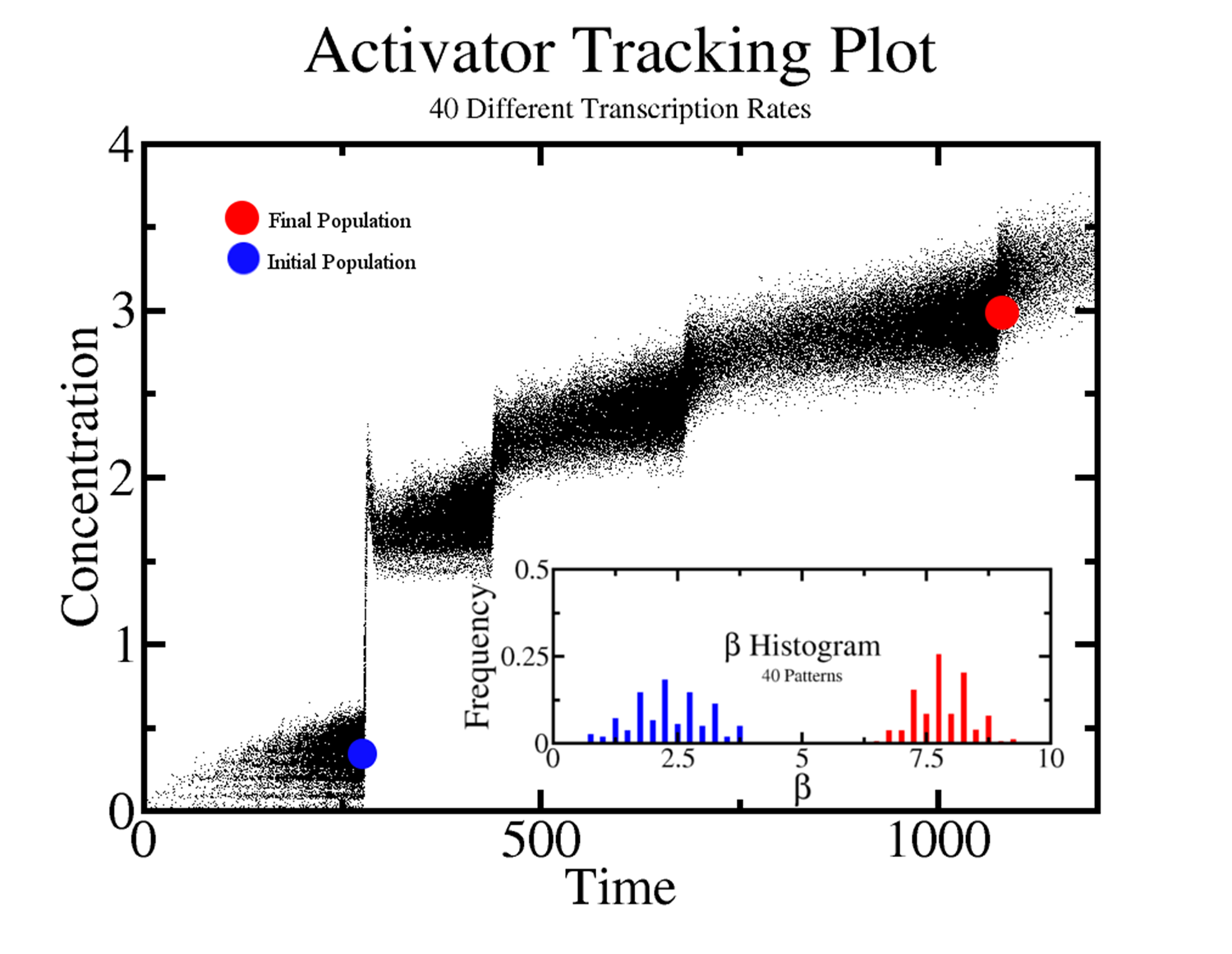

Supplement: S8 Fig — Tracking plot for the activator in the case in which β0 takes 40 different discrete values (which represent about 1% of the theoretically possible 212 different methylation patterns). In this case, in each replication the daughter cells can either acquire the same value of β0 than the mother with probability 0.5, of any of the two adjacent values with the same probability 0.25. The inset shows the distribution P(β) at the beginning of the simulation (blue histogram) and after several antibiotic shocks (red histogram). Note that the behavior of the system is essentially the same as in Fig. 2 of the main text, which indicates that a large number of methylation patterns is not necessary to obtain adaptive antibiotic resistance. (TIF) [file pone.0118464.s008.tif]

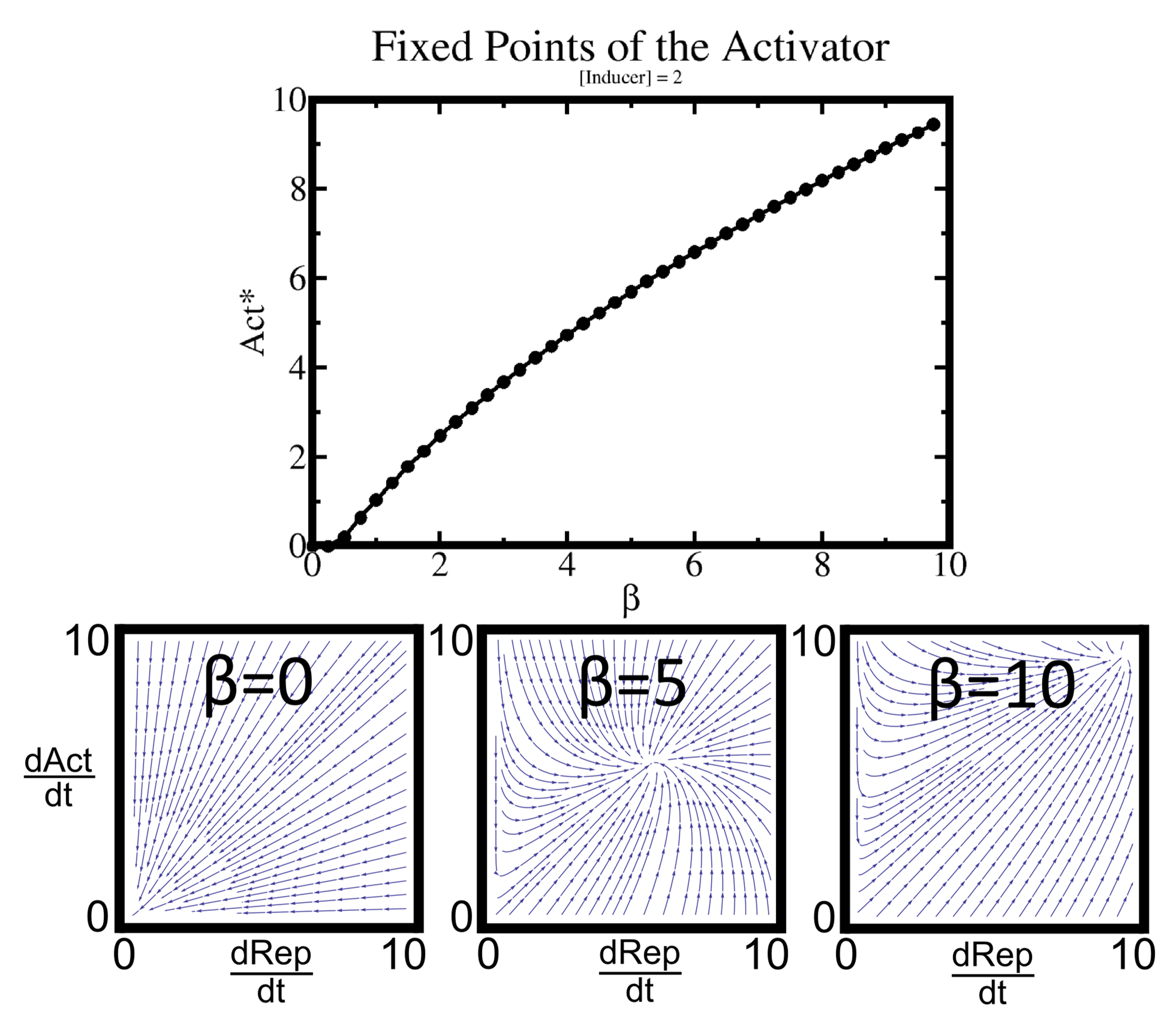

Supplement: S9 Fig — A) Fixed point of the activator as a function of β0 for a fixed concentration of external inducer. B)-D) Stream plots on the Activator-Repressor plane for different values of β0 showing that there is only one fixed point in the range of parameters explored in this work. (TIF) [file pone.0118464.s009.tif]

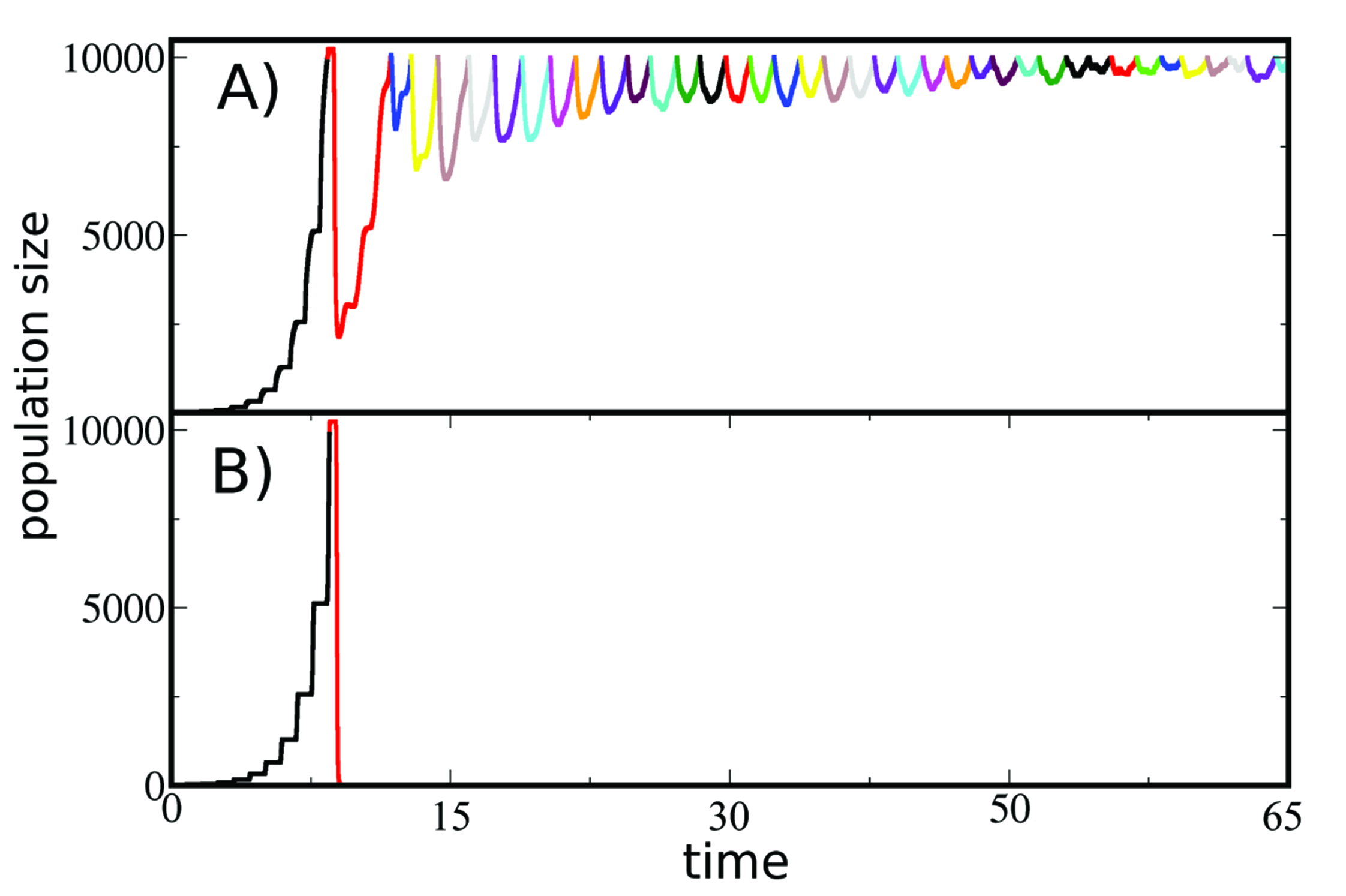

Supplement: S10 Fig — (A) Increased mutation rates. Population size for a mixed model where the genetic variability was made equal to that of the epigenetic variability, σε = σβ = 1. We can observe that the population can endure a lot more antibiotic shocks (occurring at each peak) than the mixed model where the variance in the pump efficiency was much lower 20σε = σβ (see Fig. 3B). Also, cell death is significantly reduced (approx 15% after the first induction), which is at odds with the behavior observed experimentally, where cell death is much higher [4, 5]. (B) Inverted time-scales. Population size for a mixed model where the genetic variability was interchanged with the epigenetic variability; σε = 0.1 and σβ = 0.005. We can observe that the population dies immediately after the first antibiotic shock, which supports the idea that mutations alone cannot explain adaptive resistance. Time is measured in generations. (TIF) [file pone.0118464.s010.tif]

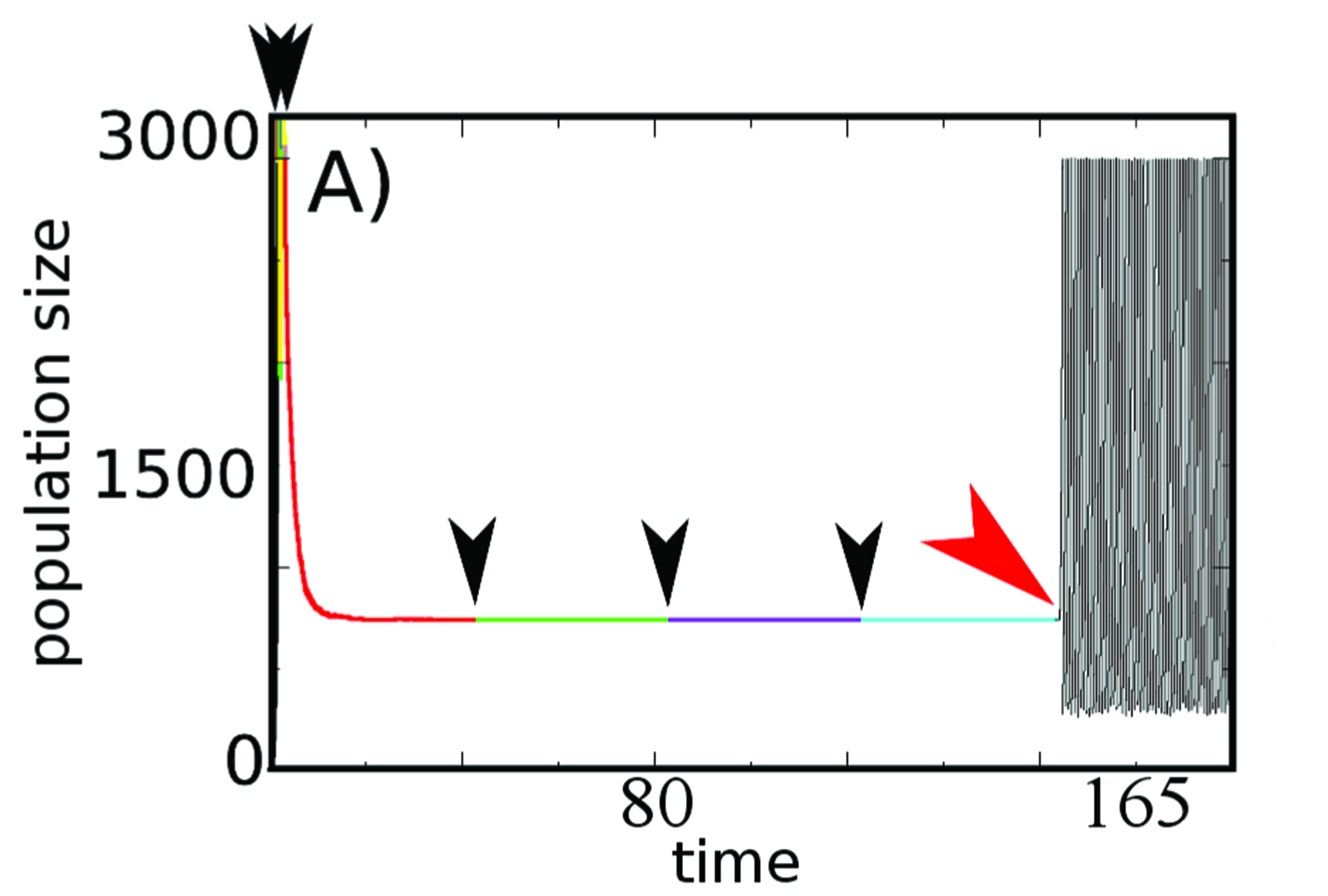

Supplement: S11 Fig — Plot of the population size as a function of time when random pump segregation is implemented without genetic or epigenetic inheritance. Each antibiotic shock is indicated by a change in color and by an arrow. Note that after the first shock just a few cells survive. These surviving cells are highly resistant because they can survive further antibiotic shocks. However, these cells cannot divide (the population size remains constant). This behavior is similar to the one observed experimentally in persistent cells [6]. (TIF) [file pone.0118464.s011.tif]

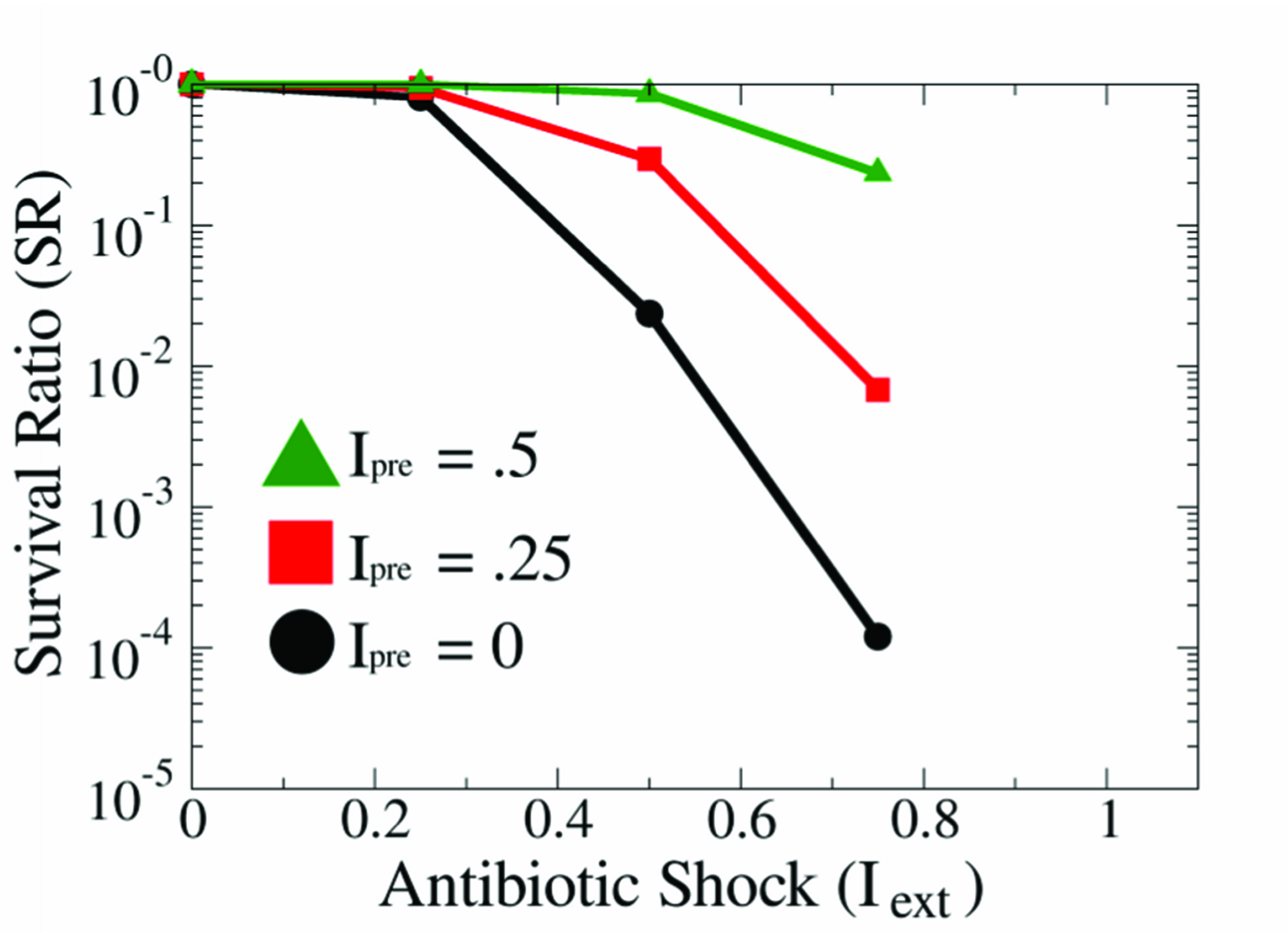

Supplement: S12 Fig — Survival ratio SR as a function of the antibiotic shock concentration Iext for different pre-induction levels: Ipre = 0 (i.e. no pre-induction, black curve), Ipre = 0.25 (red curve), and Ipre = 0.5 (green curve). Note that the survival ratio increases with the pre-induction concentration Ipre. (TIF) [file pone.0118464.s012.tif]
