# Supplementary material for: Adaptive Resistance in Bacteria Requires Epigenetic Inheritance, Genetic Noise, and Cost of Efflux Pumps
Source: PLoS One. 2015 Mar 17;10(3):e0118464. doi: 10.1371/journal.pone.0118464 (PMC4363326; doi:10.1371/journal.pone.0118464)
Supplement: S1 Table — Values of the parameters that were used in the numerical simulations of both the single-cell and population models. The second column shows the values used to obtain the results presented in the main text, while the third column shows the values used for the alternative scenarios (presented in the SI). In the latter case the values that are different from those in the original model are shaded in gray. (DOC) [file pone.0118464.s013.doc]

**Table S1. Parameter values for the evolutionary model**

| **Parameter** | **Value**  **(Original model)** | **Value (Alternative Scenarios)** |
| --- | --- | --- |
| γ_A_ | 0.5 | 0.5 |
| γ_R_ | 0.2 | 0.25 |
| γ_P_ | 0.2 | 0.5 |
| γ_Q_ | 1 | 1 |
| ι_I_ | 1 | 1 |
| ε_F_ | 0 .25 | 0.5 |
| K_R*_ | 0 .25 | 0.05 |
| K_I_ | 0 .05 | 0.05 |
| θ_F_ | 6 | 6 |
| σ_ε_ | 0 .005 | 0.01 |
| β_P_ | 0.2 | 0.3 |
| θ_I_ | 0.5 | 0.4 |
| μ_noise_ | 0 | 0 |
| μ_β_ | 0.5 | 1 |
| μ_ε_ | 0.5 | 2 |
| ϴ_diff_, ϴ_int_ | 0.1 | 0.1 |
| ρ | 1 | 1 |
| K_A_ | 1 | 1 |
| σ_noise_ | 0.1 | 0.1 |
| σ_β_ | 0 .1 | 0.25 |
| F_ext_ | 10 | 10 |
| β_Q_ | 0.1 | 0.1 |
