# Supplementary material for: Adaptive Resistance in Bacteria Requires Epigenetic Inheritance, Genetic Noise, and Cost of Efflux Pumps
Source: PLoS One. 2015 Mar 17;10(3):e0118464. doi: 10.1371/journal.pone.0118464 (PMC4363326; doi:10.1371/journal.pone.0118464)
Supplement: S2 Table — This table lists all the parameters of the model and gives their biological interpretation. (DOC) [file pone.0118464.s014.doc]

**Table S2. Meaning of the model** parameters

| A | Concentration of the Activator |
| --- | --- |
| R | Total concentration of the Repressor |
| R* | Concentration of the active form of  the Repressor |
| Q | Concentration of the Porins |
| P | Concentration of the Pumps |
| I | Internal Concentration of the Inducer |
| I_ext_ | External Concentration of Inducer |
| F | Internal Food Concentration |
| F_ext_ | External Food Concentration |
| K_I_ | Affinity of the Inducer to the Repressor |
| K_A_ | Affinity constant of the Activator |
| K_R_ | Affinity constant of the Repressor |
| β_o_ | Maximum transcription rate of the  Act-Rep operon |
| β_x_ | Maximum transcription rate of x |
| γ_x_ | Degradation rate of x |
| ρ | Entrance rate of food into the cell |
| ε_F_ | Extrusion rate of food out of the cell |
| ι_I_ | Entrance rate of inducer into the  cell |
| ε_I_ | Extrusion rate of inducer out of the  cell (Pump Efficiency) |
| μ_β_ | Average of the Gaussian determining  the transcription rate β_0_ of the  activator/repressor operon |
| σ_β_ | Variance of the Gaussian determining  the transcription rate β0 of the  activator/repressor operon |
| ξ_x_ | Gaussian noise associated to element x in the network |
| μ_noise_ | Average of the Gaussian that determines the noise ξx for each element x in the network |
| σ_noise_ | Variance of the Gaussian that determines the noise ξx for each element x in the network |
