# Supplementary material for: Adaptive Resistance in Bacteria Requires Epigenetic Inheritance, Genetic Noise, and Cost of Efflux Pumps
Source: PLoS One. 2015 Mar 17;10(3):e0118464. doi: 10.1371/journal.pone.0118464 (PMC4363326; doi:10.1371/journal.pone.0118464)
Supplement: S3 Table — Values of the parameters used for the numerical simulations of the complete Mar system depicted in Fig. 1A of the main text. (DOC) [file pone.0118464.s015.doc]

| **Complete Network Parameter** | **Value** |
| --- | --- |
| γ_Ma_ | 0.75 |
| γ_Mr_ | 0.2 |
| γ_Ar_ | 0.5 |
| γ_Aa_ | 0.25 |
| γ_Ab_ | 0.25 |
| γ_Tc_ | 0.25 |
| γ_mf_ | 0.5 |
| γ_Of_ | 0.25 |
| β_Ar_ | 0.2 |
| β_Aa_ | 0.5 |
| β_Ab_ | 0.5 |
| Β_Tc_ | 0.5 |
| Β_mf_ | .2 |
| Β_Of_ | 0.75 |
| Β_P_ | 1 |
| ι_I_ | 1 |
| ε_F_ | 0 .25 |
| K_Mr*_ | 0.5 |
| K_Ma_ | 1 |
| K_I_ | 2 |
| K_Ar_ | 0.25 |
| K_mf_ | 0.5 |

**Table S3. Parameters for the Complete Network.**
