# Supplementary material for: Adaptive Resistance in Bacteria Requires Epigenetic Inheritance, Genetic Noise, and Cost of Efflux Pumps
Source: PLoS One. 2015 Mar 17;10(3):e0118464. doi: 10.1371/journal.pone.0118464 (PMC4363326; doi:10.1371/journal.pone.0118464)
Supplement: S1 Text — (DOC) [file pone.0118464.s016.doc]

**System of differential equations of the reduced network:** The following set of equations was used to simulate the dynamics of the efflux pump regulatory network (EPRN) depicted in Fig. 1B of the main text. The values and meaning for all the variables and parameters appearing in this system of equations are given in S1_Table and S2_Table, respectively. Numerical integration was done via the fourth order Runge-Kutta method with a time step Δt = 0.001.

| $\frac{dA}{dt}=\beta_{0}\cdot\frac{A}{K_{A}+A}\cdot\frac{K_{R^{*}}}{K_{R^{*}}+R^{*}}+\beta_{1}\cdot\frac{A}{K_{A}+A}-\gamma_{A}A+\xi_{A}$ |  | (1) |
| --- | --- | --- |
| $\frac{dR}{dt}=\beta_{0}\cdot\frac{A}{K_{A}+A}\cdot\frac{K_{R^{*}}}{K_{R^{*}}+R^{*}}-\gamma_{R}R+\xi_{R}$ |  | (2) |
| $\frac{dQ}{dt}=\beta_{Q}\cdot\frac{K_{A}}{K_{A}+A}-\gamma_{Q}Q+\xi_{Q}$ |  | (3) |
| $\frac{dP}{dt}=\beta_{P}\cdot\frac{A}{K_{A}+A}-\gamma_{P}P+\xi_{P}$ |  | (4) |
| $\frac{dI}{dt}=\iota_{I}\cdot Q(I_{ext}-I)-(\varepsilon_{I}\cdot P\cdot I)+\xi_{I}$ |  | (5) |
| $\frac{dF}{dt}=\rho\cdot\frac{F_{ext}-F}{\theta_{diff}+(F_{ext}-F)}-\varepsilon_{F}\cdot\frac{P\cdot F}{\theta_{F}+F}+\xi_{F}$ |  | (6) |
| $R^{*}=\frac{R}{1+\frac{I}{K_{I}}}$ |  | (7) |

Note that the last equation is algebraic and relates the active form of the repressor (R*) with the total amount (R). We will refer to the set of equations above as the single-cell EPRN (SC-EPRN).

**System of differential equations for the complete network:** The following set of equations was used to simulate the dynamics of the complete Mar system shown in Fig. 1A of the main text. The values of the parameters in this system of equations are given in S3_Table. Numerical integration was done via the fourth order Runge-Kutta method with a time step Δt = 0.001. A comparison of the dynamics of complete and simplified networks can be observed in S1_Fig.

$$\frac{dMarA}{dt}=\beta_{0}\cdot\frac{MarA}{K_{Ma}+MarA}\cdot\frac{K_{Mr}}{K_{Mr}+MarR^{*}}\beta_{1}\cdot\frac{MarA}{K_{Ma}+MarA}-\gamma_{Ma}MarA+\xi_{Ma}$$

$$\frac{dMarR}{dt}=\beta_{0}\cdot\frac{MarA}{K_{Ma}+MarA}\cdot\frac{K_{Mr}}{K_{Mr}+MarR^{*}}-\gamma_{Mr}MarR+\xi_{Mr}$$

$$\frac{dAcrR}{dt}=\beta_{Ar}-\gamma_{Ar}AcrR+\xi_{Ar}$$

$$\frac{dAcrA}{dt}=\beta_{Aa}\cdot\frac{MarA}{K_{Ma}+MarA}\cdot\frac{K_{Ar}}{K_{Ar}+AcrR}-\gamma_{Aa}AcrA+\xi_{Aa}$$

$$\frac{dAcrB}{dt}=\beta_{Ab}\cdot\frac{MarA}{K_{Ma}+MarA}\cdot\frac{K_{Ar}}{K_{Ar}+AcrR}-\gamma_{Ab}AcrB+\xi_{Ab}$$

$$\frac{dTolC}{dt}=\beta_{Tc}\cdot\frac{MarA}{K_{Ma}+MarA}-\gamma_{Tc}TolC+\xi_{Tc}$$

$$\frac{dmicF}{dt}=\beta_{mf}\cdot\frac{MarA}{K_{Ma}+MarA}-\gamma_{mf}micF+\xi_{mf}$$

$$\frac{dOmpF}{dt}=\beta_{Of}-(K_{mf}\cdot OmpF\cdot micF)-\gamma_{Of}OmpF+\xi_{Of}$$

$$\frac{dI}{dt}=\iota_{I}\cdot OmpF(I_{ext}-I)-(\varepsilon_{I}\cdot Pumps\cdot I)+\xi_{I}$$

$$\frac{dF}{dt}=\rho\cdot\frac{F_{ext}-F}{\theta_{diff}+(F_{ext}-F)}-\frac{\varepsilon_{F}\cdot Pumps\cdot F}{\theta_{F}+F}+\xi_{F}$$

$$\frac{dPumps}{dt}=\beta_{P}\cdot(\frac{AcrA^{2}\cdot AcrB\cdot TolC}{\omega_{p}})$$

$$MarR^{*}=\frac{MarR}{1+\left(\frac{I}{K_{I}}\right)^{2}}$$

**Single Cell Experiments:** S2_Fig reports the behavior of the SC-EPRN. In particular, it shows the ratio Г_x_ = X_a_/X_w_ between the stationary expression level of element X in the network with antibiotic (X_a_), and without antibiotic (X_w_). It can be observed that the introduction of an external concentration of antibiotic yields an increase in the concentration of the activator (ГA > 1) which in turn induces the production of efflux pumps (Г_p_ > 1). Also the total amount of repressor increases (Г_R_ > 1) because it gets transcribed in the same transcription unit as the activator, but its active form is greatly decreased (Г_R*_ < 1) as the antibiotic binds to it and inactivates it. The results presented in S2_Fig indicate that the SC-EPRN behaves as expected for wild type cells. Nonetheless, we further validate it against published expression levels in a mutant strain. (In our numerical experiments mutants with single gene deletions were simulated by setting to zero the concentration of the deleted element throughout the simulation time.) We illustrate this by considering the known behavior of a mutant whose efflux channel has been corrupted [1]. For this mutant it is known that with a defective channel of the efflux pumps the concentration of the activator increases as toxic substances accumulate inside the cell. This further promotes the inactivation of the repressor which increases the transcription rate of the activator due to its positive auto regulation. In S3_Fig we show the expression level of the activator as a function of time for wild type (P^+^) and pump deficient strains (P^-^). Both realizations were carried out with an external inducer concentration I_ext_ = 3. Experimental data reported in [1] show a twofold increase in the concentration of the activator in the pump defective strain with respect to the wild type. This observation helped us to further calibrate some of the parameters of the single cell model which, as can be observed in S3_Fig, yields also a twofold increase (approximately) in the expression of the activator when the efflux pumps are inactivated.

**Parameter Variation:** In the main article we used of a set of parameters that qualitatively reproduce various experiments related with adaptive resistance. Nonetheless, it is necessary to test the robustness of the model under changes in the parameters. It is worth noting that the transcription rate β_0_ and the pump efficiency ε_I_ do constantly change during the simulation, as they are the targets of variability and inheritance. However, all the other parameters remained fixed during the simulation. An exhaustive search of the regions in the parameter space that yield the same qualitative results as the ones presented in the main text is far beyond the scope of this paper. Nonetheless, we performed numerical experiments where some of the parameters changed even in one order of magnitude with no qualitative change in the results. Here we present an example (S4_Fig and S5_Fig) where three parameters are varied within a finite range with no significant change in the induction experiments. These three parameters are: The degradation rate of the Activator (γ_A_), the degradation rate of the repressor (γ_R_) and the average increase of the antibiotic concentration between two successive shocks. By moving these parameters along the curve shown in S4_Fig we obtain the same qualitative behavior of the activator concentration in the population, as S5_Fig shows.

**Continuous uniform distribution for β**. S6_Fig shows the results of a numerical simulation in which β_0_ is uniformly distributed between 0 and 10. In this case, there is no correlation between the mother and the daughter cells. At each generation, each cells simply takes a random value in the interval [0,10] with uniform probability. It is apparent from this figure that without mother-daughter correlations, the population cannot withstand antibiotic inductions even in the presence of high variability.

**Energy Sources and Cell Division Rates**. It has been observed experimentally that the concentration of inducer directly affects the cell division rate. Indeed, the higher the antibiotic levels a cell is able to resist, the longer the time it takes for the cell to divide [2]. The SC-EPRN presented here reproduces this behavior, as S7_Fig shows, where it is apparent that the average cell division time increases with both the transcription rate β_0_ and the external antibiotic concentration I_ext_. The reason for this increase is twofold. First, increasing the transcription rate β_0_ also increases the production of pumps, even in the absence of antibiotic. Second, the introduction of the antibiotic allows the activator to promote its own transcription (through inhibition of the repressor), which further increases the production of pumps. In both cases, having more pumps increases the division time because the pumps do not only pump out the antibiotic, but also nutrients. Thus, S7_Fig shows that the energetic burden conveyed by the production of efflux pumps is enough to qualitatively reproduce the lag in the division time observed when the cells become more and more resistant.

**Discretized distribution for β.** In the simulations shown in S8_Fig, the interval [0,10] was discretized into 40 values for β_0_ (so that β_0_ = n/4 with n = 0,1, 2,…,40). The inheritance mechanisms worked as follows. An initial population of 12 cells was generated, in which 6 cells had β_0_ = 0.5, 3 cells had β_0_ = 0.25 and the other 3 cells had β_0_ = 0.75. In each generation each cell replicates until a population of 5000 cells is reached. In each replication, the daughter cells will correlate their values of β_0_ with that of the mother cell according to the following rule. Let β_0_(*g*)= *n_g_*/4 the value of the transcription rate of the mother cell at generation *g*. Then, the value of β_0_(*g+*1) for each daughter cell at generation *g*+1 is given by:

β_0_(*g+*1) = (*n_g_* + 1)/4 with probability 0.25

β_0_(*g+*1) = (*n_g_* - 1)/4 with probability 0.25

β_0_(*g+*1) = *n_g_*/4 with probability 0.5

Thus, with probability 0.5 each daughter cell takes the same value of β_0_ than the mother, and with probability 0.5 it takes the adjacent value either to the right or to the left.

S8_Fig shows the tracking plots of the activator for a simulation following successive inducer concentrations in this case where β_0_ takes the discrete values mentioned above. Comparing S8_Fig with Fig. 2 of the main text, it can be observed that in both cases, (i.e. a discrete distribution or a continuous Gaussian distribution for β_0_), the same qualitative results are obtained.

**Fixed Points.** The fixed points for the system of differential equations describing the simplified EPRN were calculated. S9A_Fig shows how the fixed point of the activator changes with β_0_, whereas the other panels in this figure show the projection of the fixed point on the Activator-Repressor plane for three different values of β. It can be seen from this figure that there is only one fixed point in the region of the parameter space that we explored in this manuscript. Therefore, changes in the dynamical behavior of the system are not produced by the system “jumping” between different fixed points. Rather, the emergence of adaptive resistance has to do with a gradual shift of the fixed point with β_0_ as the system is subjected to external stress and selection.

**Alternative Scenarios:** Apart from the epigenetic mechanism presented in the main text to generate adaptive resistance, we have also explored other scenarios, which may appear biologically plausible. For instance, an increased mutation rate has been previously suggested as a cause. Although it is experimentally known that mutation rates can explain neither the gradual emergence of the resistance phenotype nor its fast reversibility, we wanted to see the behavior of the population under this particular situation.

For this, we have tried two different scenarios for the Mixed inheritance model.

1. We have set both variances to the upper value max(σ_β_, σ_ε_)
2. We have switched the values of the variances σ_ε_ and σ_β_ , so σ_ε >>_ σ_β_

S10A_Fig shows the results for the first scenario. As it can be observed the population acquires resistance at a very fast pace and with very low death rates (compare with the mixed scenario discussed in the main article, Fig. 3B). This was expected, in the sense that the probability for having a very high pump efficiency was increased, and added to the probability of producing a considerable amount of efflux pumps. Therefore, with this amount of genetic variability added to epigenetic variability, significant changes improving the resistant phenotype arise so quickly that the population becomes immediately and almost completely, resistant to the antibiotic.

S10B_Fig shows the second scenario where the effect of the epigenetic variability is minimized and the genetic variability maximized. Here, the population is not able to survive more than one antibiotic shock, supporting the hypothesis that genetic mutation alone, even at high rates, cannot explain the emergence of adaptive resistance.

Another plausible scenario for adaptive resistance to occur is the transmission of active pumps from mother to daughter cells. It is known that very stable proteins lasting for more than one cell division are transmitted to both cells after cell division. If this were the case for the efflux pumps, the daughter cells would have more pumps than the mother had at birth. Therefore, the daughter cells would exhibit an increased level of resistance than the mother. Additionally, if the distribution of pumps between the daughter cells is biased or uneven [3] then one daughter cell would receive more pumps than the other. Consequently, the daughter cell with more pumps will show a more resistant phenotype than the one with less pumps. Thus, transmission of active pumps from the mother to the daughter cells, along with an uneven distribution of pumps between the daughter cells, could constitute a trivial explanation of the variability of the resistant phenotype among isogenic cells and the inevitable emergence of highly resistant cells (the ones that just by chance have accumulated a considerable excess of pumps). Although there is evidence that some efflux pumps form clusters in the membrane [3], to our knowledge there is no concrete evidence showing that efflux pumps segregate unevenly through the cell membrane and that this uneven segregation is the cause of the emergence of highly resistant cells. We implemented in our model the following three scenarios of efflux pump segregation between daughters in order to determine whether or not the mechanism described above can indeed explain the gradual emergence of the resistant phenotype:

1. Random segregation. The concentration of pumps at each cell division is partitioned by a random number *r* from the uniform distribution between 0 and 1. In each cell division one daughter cell receives a fraction r of the pumps of the mother while the other cell receives the complementary fraction 1 − r.
2. Biased segregation 90%-10%. Here, at each cell division one daughter cell receives 90% of the pumps of the mother, and the other receives only 10%. The decision as to which of the two daughter cells receives more pumps is made randomly with probability 1/2.
3. Even segregation 50%-50%. In this case the pumps of the mother cell are equally partitioned between the two daughters at each cell division.

The above segregation scenarios were implemented in numerical simulations similar to the ones described in the main text (like in Fig. 2) and with the same parameters (given in S1_Table), but eliminating both types of inheritance (genetic and epigenetic). Interestingly, with these parameters none of the segregation scenarios produced population able to survive beyond the first two antibiotic shocks. Actually, most of the cells died with the very first shock. Therefore, we performed a thorough search in the parameter space in order to get one set of parameters for which some cells survived at least the first two rounds of induction. This search produced the set of parameters shown in the third column of S1_Table. S11_Fig shows the size of the population as a function of time for the random pump segregation case with the alternative parameters listed in S1_Table (and with neither genetic nor epigenetic inheritance). It is apparent from this figure that the random segregation of pumps alone cannot explain the emergence of the resistant phenotype as observed experimentally. For as soon as the antibiotic is added to the media, most of the cells in the population die, with the exception of a few surviving cells. However, contrary to what happens with adaptive resistance, the surviving cells in this new case are “super resistant” in the sense that, regardless of the amount of antibiotic that was introduced into the media, they were able to cope with it. Nonetheless, these surviving cells are not able to go through cell division due to the high energy consumption imposed by the production of pumps. These “persistent cells” (i.e. highly resistant to antibiotics but unable to divide) have been observed experimentally [4, 5, 6]. A similar situation happens with the biased and even segregation mechanisms.

**Preinduction Experiments.** It is known experimentally that cells that have been pre-induced with non-lethal concentrations of antibiotics exhibit higher resistance to later antibiotic shocks than those who have not been pre-induced [7]. (In fact, this is one of the reasons for the antibiotic restriction program in public health facilities [8].) To see if such behavior is predicted by our model, we started with a population of 10 cells and let them divide in a media with a low concentration of external antibiotic I_pre_ until a population size of 100 cells was reached. At this point the population was subjected to an antibiotic shock I_ext_ above the lethal concentration and the survival ratio was measured. S12_Fig reports the survival ratio as a function of the antibiotic shock concentration for three different preinduction levels (including no preinduction) and five different antibiotic shocks. By comparing our results with experimental data [7] we can observe that our model reproduces this feature qualitatively. Clearly, when there is no variability in the population, namely when all the cells have the same values for the parameters β_0_ and ε_I_ , an all-or-none phenomenon is observed in the pre-induction experiments: for small βo all the cells die with the first antibiotic shock, whereas for large βo all the cells survive several rounds of antibiotic shocks. This behavior is interesting because it occurs even in the presence of stochastic noise (modeled by the variables ξ_x_ in Eqs. 1), which illustrates that stochastic noise is irrelevant for the emergence of the resistant phenotype.

**References**

1. Rosner JL, Martin RG. An excretory function for the Escherichia coli outer membrane pore TolC: upregulation of marA and soxS transcription and Rob activity due to metabolites accumulated in tolC mutants. J Bacteriol. 2009;191(16):5283-5292. doi:10.1128/JB.00507-09.
2. Oethinger M, Podglajen I, Kern W V, Levy SB. Overexpression of the marA or soxS regulatory gene in clinical topoisomerase mutants of Escherichia coli. Antimicrob Agents Chemother. 1998;42:2089-2094.
3. Lau SY, Zgurskaya HI. Cell division defects in Escherichia coli deficient in the multidrug efflux transporter AcrEF-TolC. J Bacteriol. 2005;187:7815-7825. doi:10.1128/JB.187.22.7815-7825.2005.
4. Cohen NR, Lobritz MA, Collins JJ. Microbial persistence and the road to drug resistance. Cell Host Microbe. 2013;13:632-642. doi:10.1016/j.chom.2013.05.009.
5. Kussell E, Kishony R, Balaban NQ, Leibler S. Bacterial persistence: a model of survival in changing environments. Genetics. 2005;169:1807-1814. doi:10.1534/genetics.104.035352.
6. Andersson DI, Hughes D. Persistence of antibiotic resistance in bacterial populations. FEMS Microbiol Rev. 2011;35:901-911. doi:10.1111/j.1574-6976.2011.00289.x.
7. Adam M, Murali B, Glenn NO, Potter SS. Epigenetic inheritance based evolution of antibiotic resistance in bacteria. BMC Evol Biol. 2008;8:52. doi:10.1186/1471-2148-8-52.
8. Siddiqui S, Hussein K, Manasia R, Samad A, Salahuddin N, et.al. Impact of antibiotic restriction on broad spectrum antibiotic usage in the ICU of a developing country. J Pak Med Assoc. October 2007. Vol. 57, No. 10.
